# Supplementary material for: A randomization-based causal inference framework for uncovering environmental exposure effects on human gut microbiota
Source: PLoS Comput Biol. 2022 May 9;18(5):e1010044. doi: 10.1371/journal.pcbi.1010044 (PMC9129050; doi:10.1371/journal.pcbi.1010044)
Supplement: S1 Text — Fig A: Gut microbiome data description. Number of observed ASV per sample (top left), sequencing depth per sample (top right), number of sequences per ASV (bottom left), number of zero count per ASV (bottom right). Fig B: Empirical distributions of the matched covariates among the subjects under the intervention vs. not in the original (left panel) and the balanced (right panel) data for the air pollution reduction hypothetical experiment. Fig C: Empirical distributions of the disease covariates among the subjects under the intervention vs. not in the original (left panel) and the balanced (right panel) data for the air pollution reduction hypothetical experiment. Fig D: Empirical distributions of the medication covariates among the subjects under the intervention vs. not in the original (left panel) and the balanced (right panel) data for the air pollution reduction hypothetical experiment. Fig E: Empirical distributions of the matched covariates among the subjects under the intervention vs. not in the original (left panel) and the balanced (right panel) data for the smoking prevention hypothetical experiment. Fig F: Empirical distributions of the diseases covariates among the subjects under the intervention vs. not in the original (left panel) and the balanced (right panel) data for the smoking prevention hypothetical experiment. Fig G: Empirical distributions of the medication covariates among the subjects under the intervention vs. not in the original (left panel) and the balanced (right panel) data for the smoking prevention hypothetical experiment. Fig H: Empirical distributions of the nutrition covariates among the subjects under the intervention vs. not in the balanced data for the air pollution reduction hypothetical experiment. Fig I: Empirical distributions of the nutrition covariates among the subjects under the intervention vs. not in the balanced data for the smoking prevention hypothetical experiment. Fig J: Permutation-based (grey) and asymptotic (bl [file pcbi.1010044.s001.pdf]

Supplementary material: A randomization-based causal inference framework for uncovering environmental exposure effects on human gut microbiota

Alice J Sommer[1,2,3,*], Annette Peters[2,3,4,*], Martina Rommel[3,5], Josef Cyrys[3], Harald Grallert[5,6], Dirk Haller[7,8], Christian L Müller[9,10,11,*], and Marie-Abèle C Bind[1,12]

[1]Department of Statistics, Harvard University, Cambridge, MA, USA
[2]Institute for Medical Information Processing, Biometry, and Epidemiology, Faculty of Medicine, Ludwig-Maximilians-University München, Munich, Germany
[3]Institute of Epidemiology, Helmholtz Zentrum München, Neuherberg, Germany
[4]Department of Environmental Health, Harvard T. H. Chan School of Public Health, Boston, MA, USA
[5]Research Unit of Molecular Epidemiology, Helmholtz Zentrum München, Neuherberg, Germany
[6]German Center for Diabetes Research (DZD), München-Neuherberg, Germany
[7]ZIEL - Institute for Food & Health, Technical University of Munich, Freising, Germany
[8]Chair of Nutrition and Immunology, Technical University of Munich, Freising, Germany
[9]Institute of Computational Biology, Helmholtz Zentrum München, Neuherberg, Germany
[10]Department of Statistics, Ludwig-Maximilians-University München, Munich, Germany
[11]Center for Computational Mathematics, Flatiron Institute, New York, NY, USA
[12]Biostatistics Center, Massachusetts General Hospital and Harvard Medical School, Boston, MA, USA
[*]Corresponding authors: Alice J. Sommer: alice.j.sommer@gmail.com,
Annette Peters: peters@helmholtz-muenchen.de, and Chrisitan L. Müller: cmueller@flatironinstitute.org

-

Gut microbiome data description (Amplicon Sequence Variants)

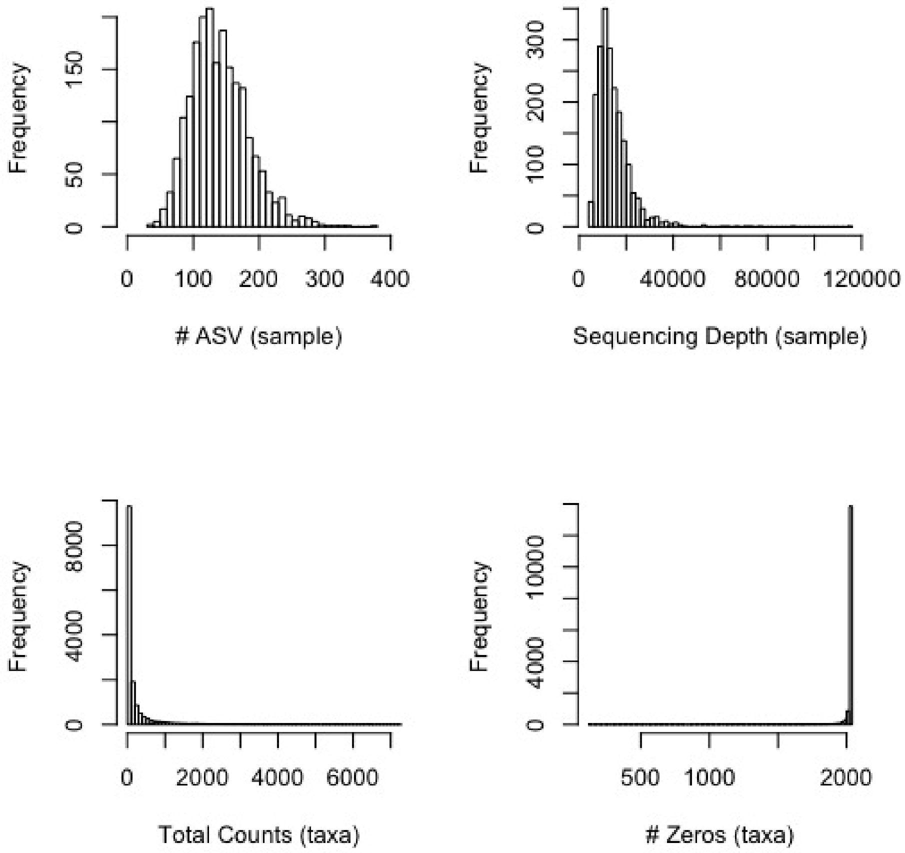

Fig A. Gut microbiome data description. Number of observed ASV per sample (top left), sequencing depth per sample (top right), number of sequences per ASV (bottom left), number of zero count per ASV (bottom right).

	Min.	1st Qu.	Median	Mean	3rd Qu.	Max.
nb. ASV (per sample)	31	109	135	140	168	371
nb. counts (per sample)	4,696	9,696	12,716	14,470	17,292	115,055
nb. counts (per taxa)	1	16	61	1,863	219	729,636
nb. zeros (per taxa)	122	2,030	2,033	2,016	2,033	2,033

Table A. Gut microbiome data description. Number of observed ASV per sample, sequencing depth per sample, number of sequences per ASV, number of zero count per ASV.

Balance diagnostics for matching

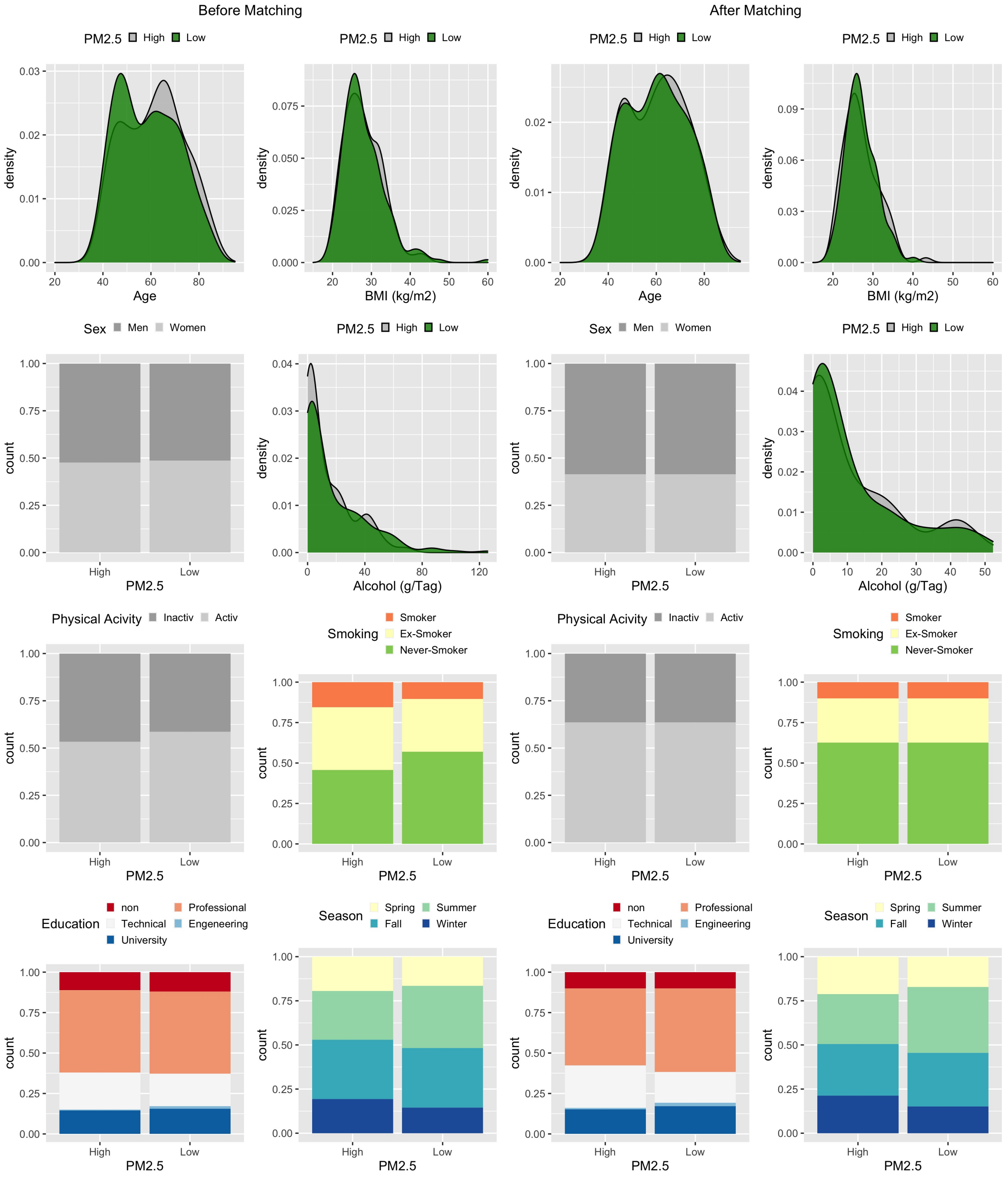

Fig B. Empirical distributions of the covariates among the subjects under the intervention vs. not in the original (left panel) and the balanced (right panel) data for the air pollution reduction hypothetical experiment.

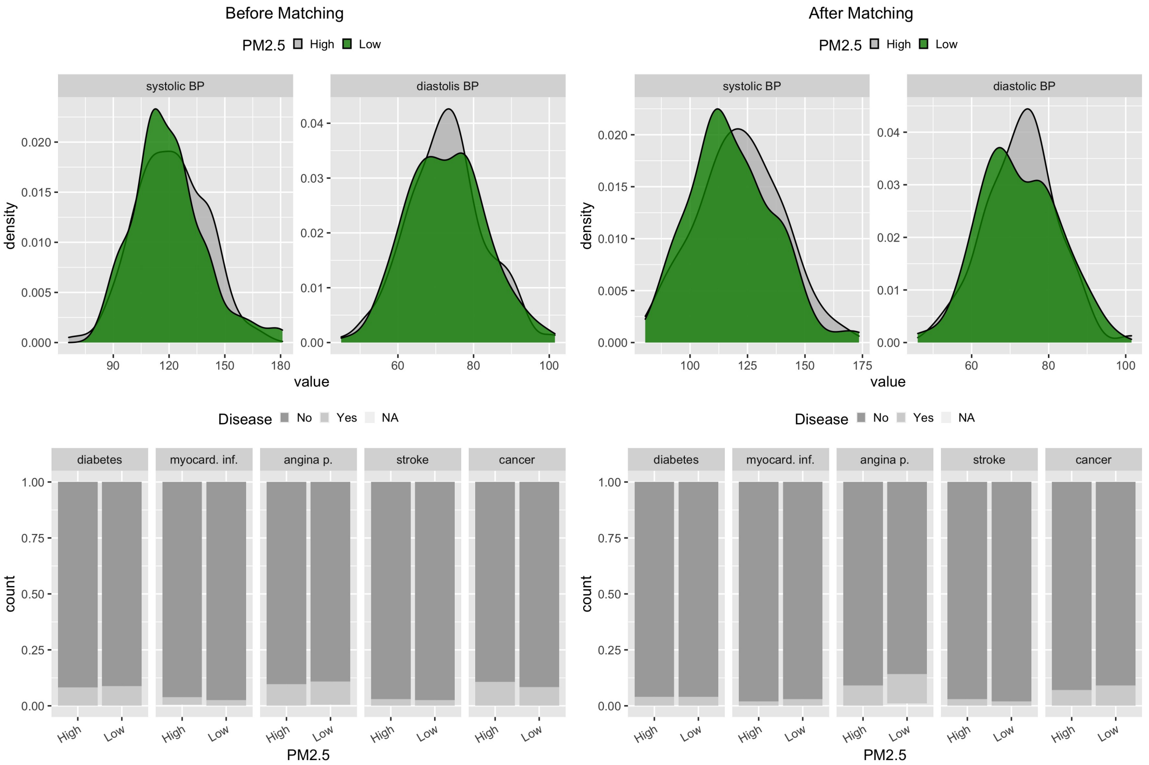

Fig C. Empirical distributions of the disease covariates among the subjects under the intervention vs. not in the original (left panel) and the balanced (right panel) data for the air pollution reduction hypothetical experiment.

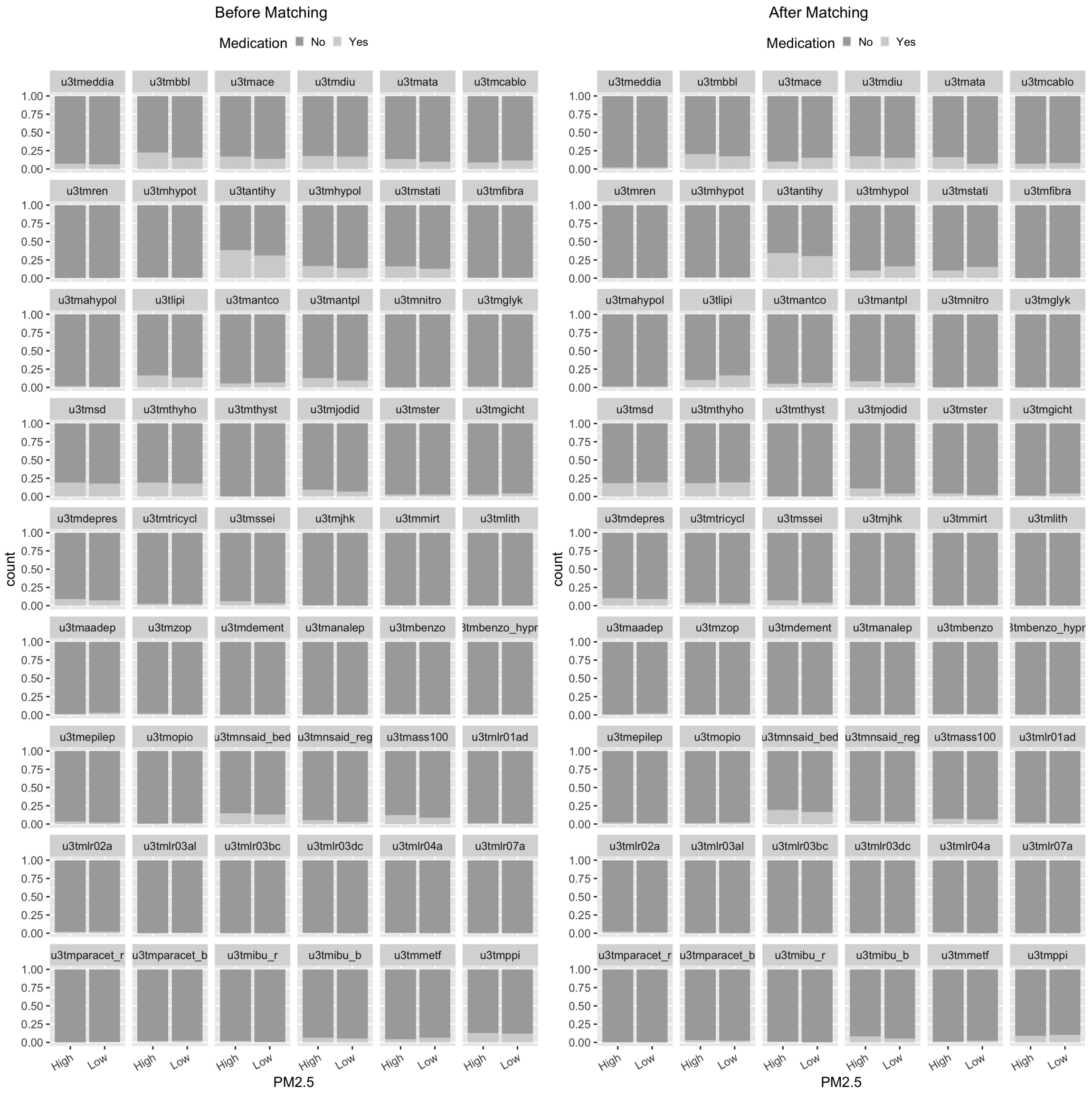

Fig D. Empirical distributions of the medication covariates among the subjects under the intervention vs. not in the original (left panel) and the balanced (right panel) data for the air pollution reduction hypothetical experiment.

Smoking

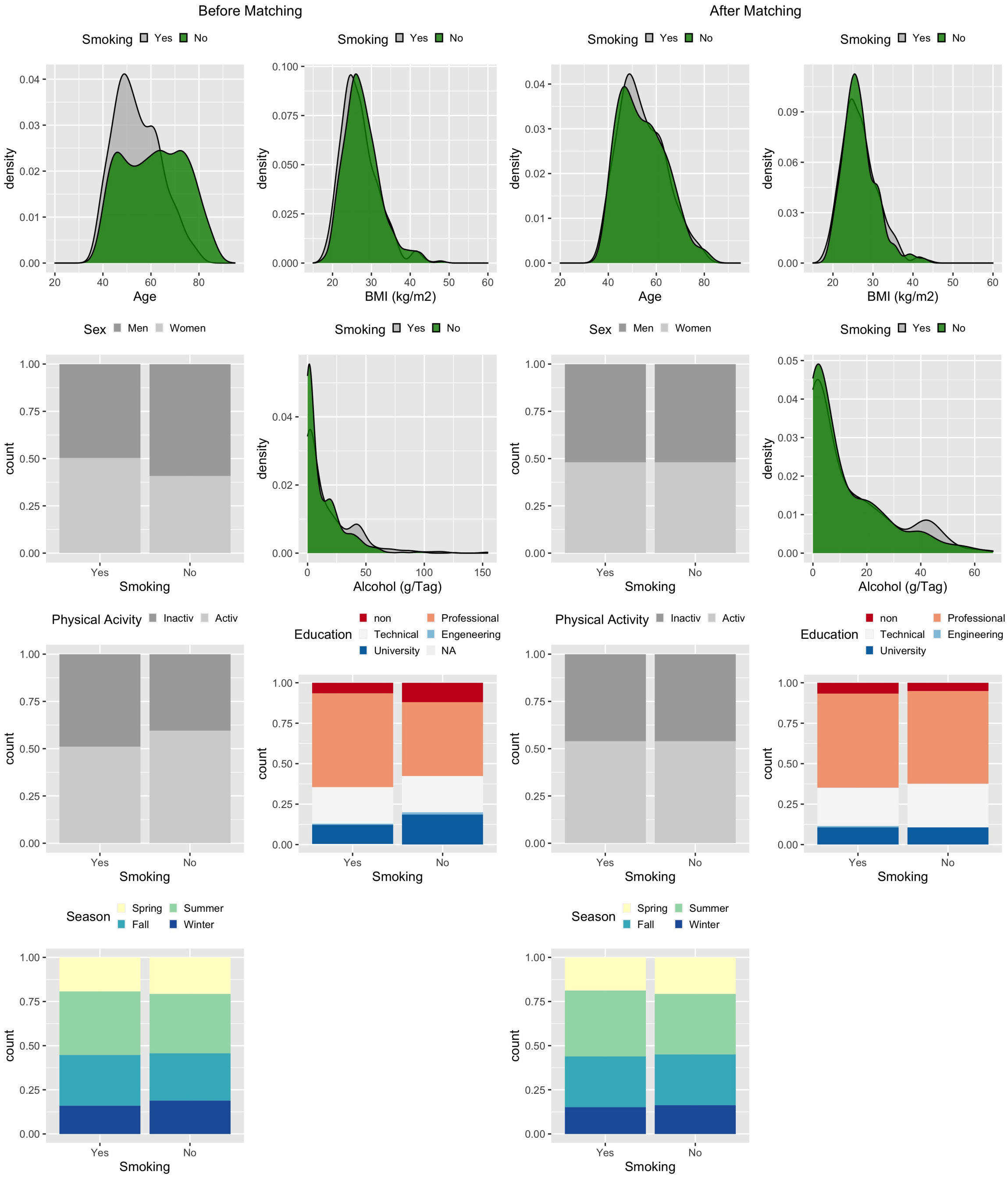

Fig E. Empirical distributions of the covariates among the subjects under the intervention vs. not in the original (left panel) and the balanced (right panel) data for the smoking prevention hypothetical experiment.

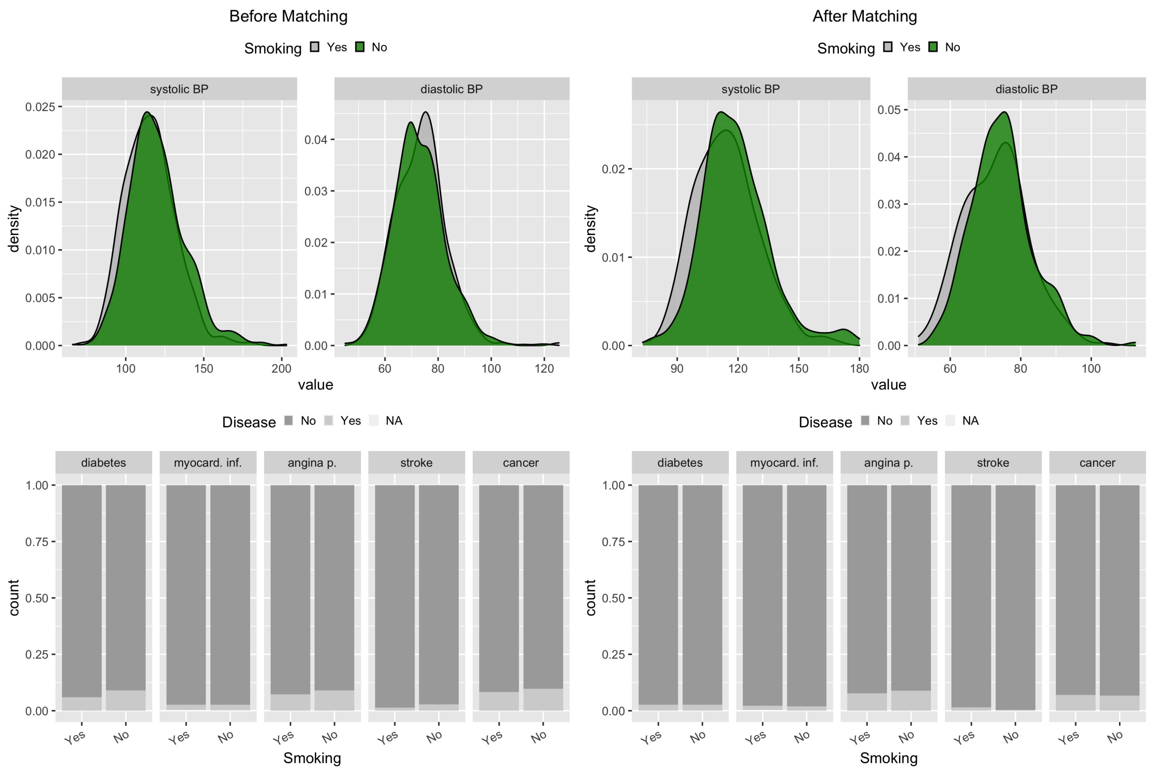

Fig F. Empirical distributions of the diseases covariates among the subjects under the intervention vs. not in the original (left panel) and the balanced (right panel) data for the smoking prevention hypothetical experiment.

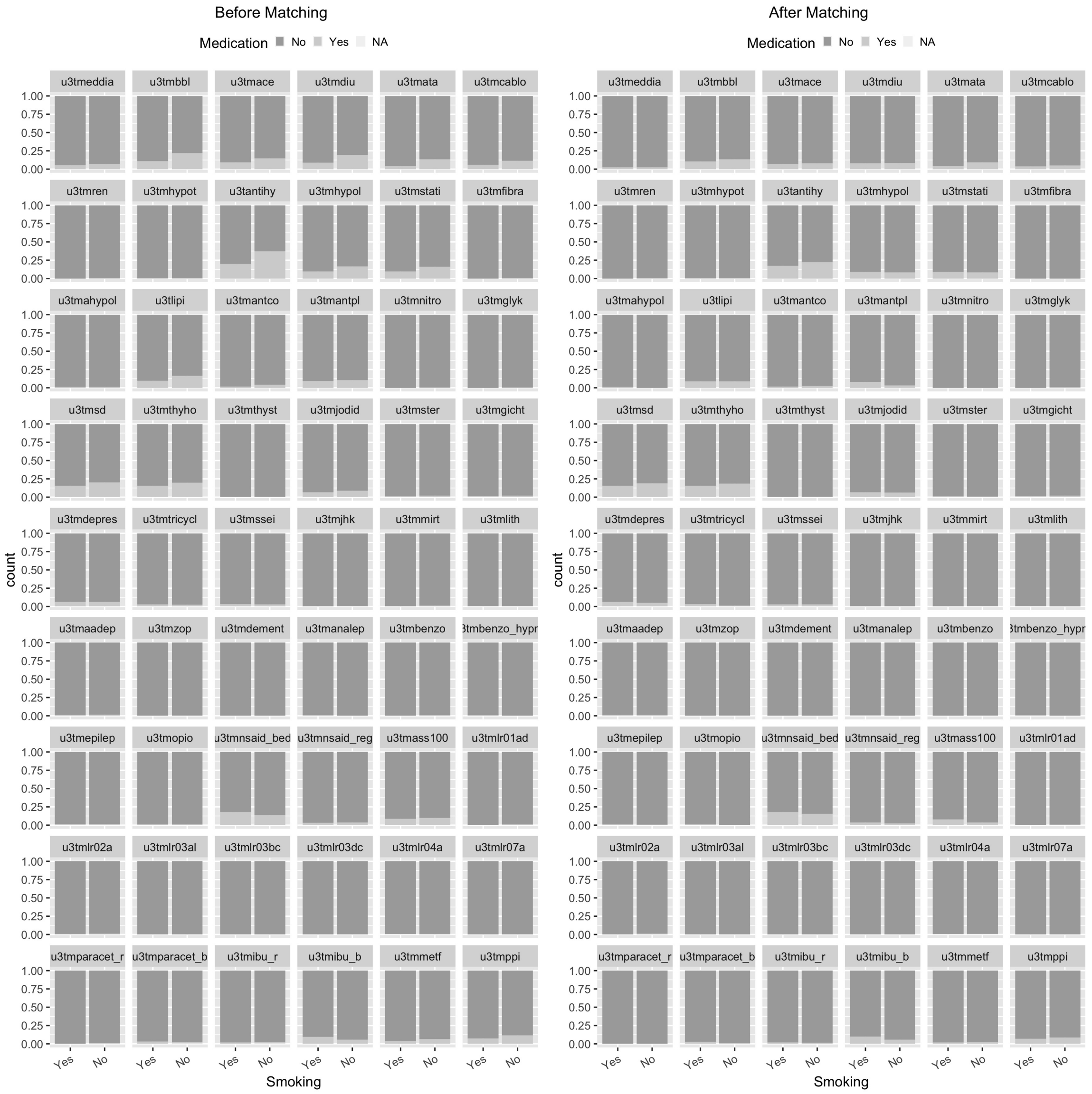

Fig G. Empirical distributions of the medication covariates among the subjects under the intervention vs. not in the original (left panel) and the balanced (right panel) data for the smoking prevention hypothetical experiment.

Balance diagnostics for nutrition covariates after matching

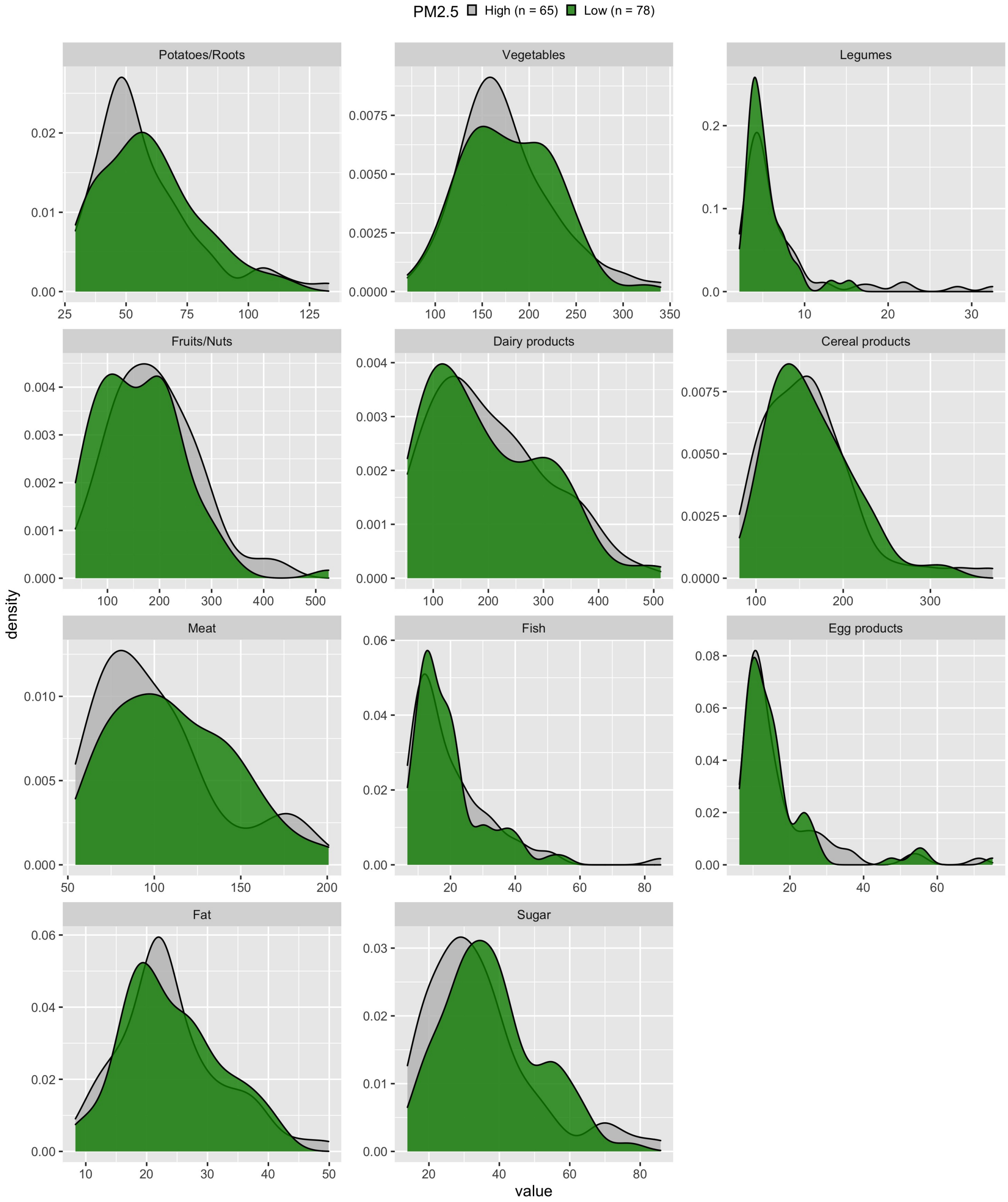

Fig H. Empirical distributions of the nutrition covariates among the subjects under the intervention vs. not in the balanced data for the air pollution reduction hypothetical experiment.

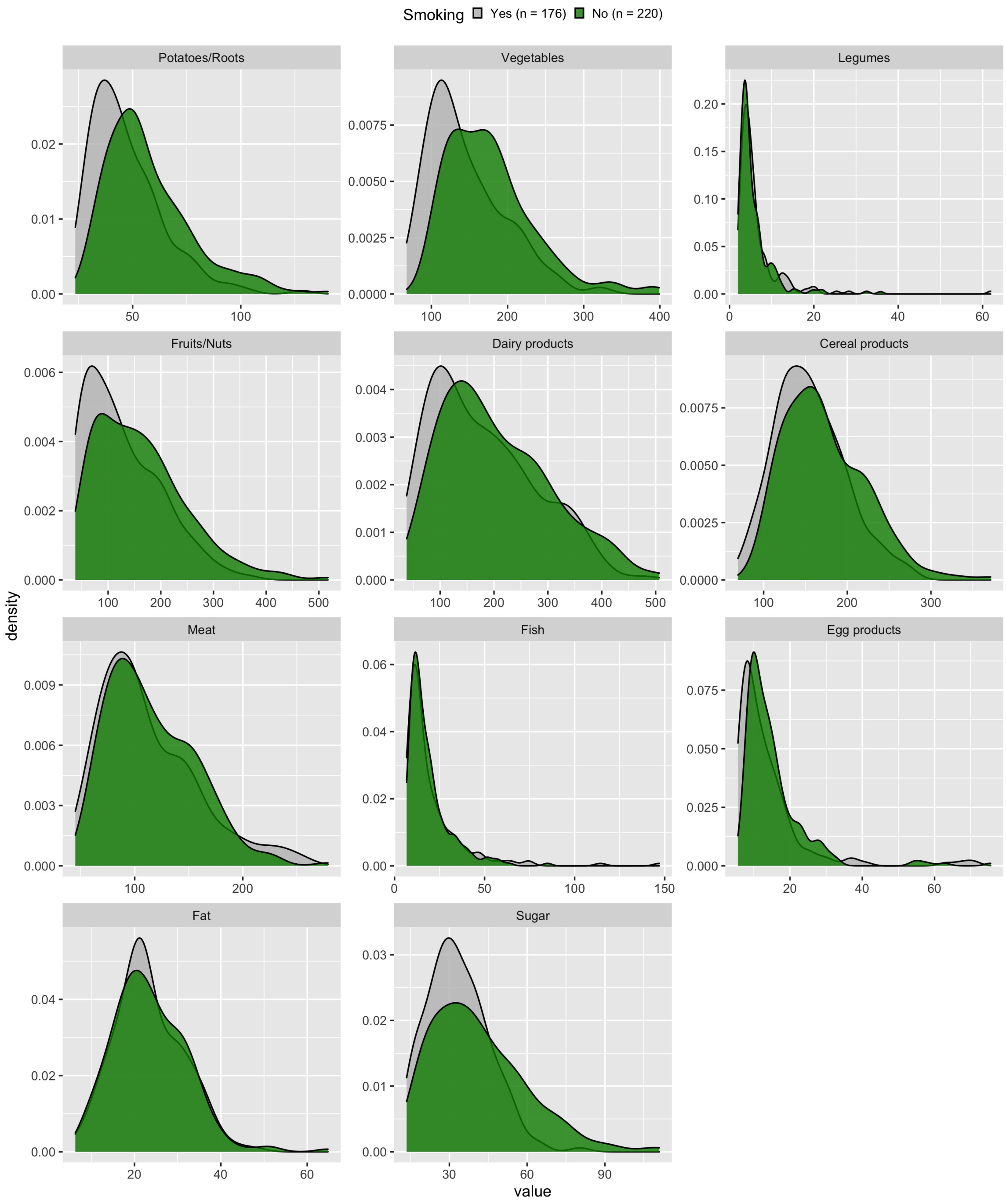

Fig I. Empirical distributions of the nutrition covariates among the subjects under the intervention vs. not in the balanced data for the smoking prevention hypothetical experiment.

Comparison of permutation and asymptotic null randomization distribution

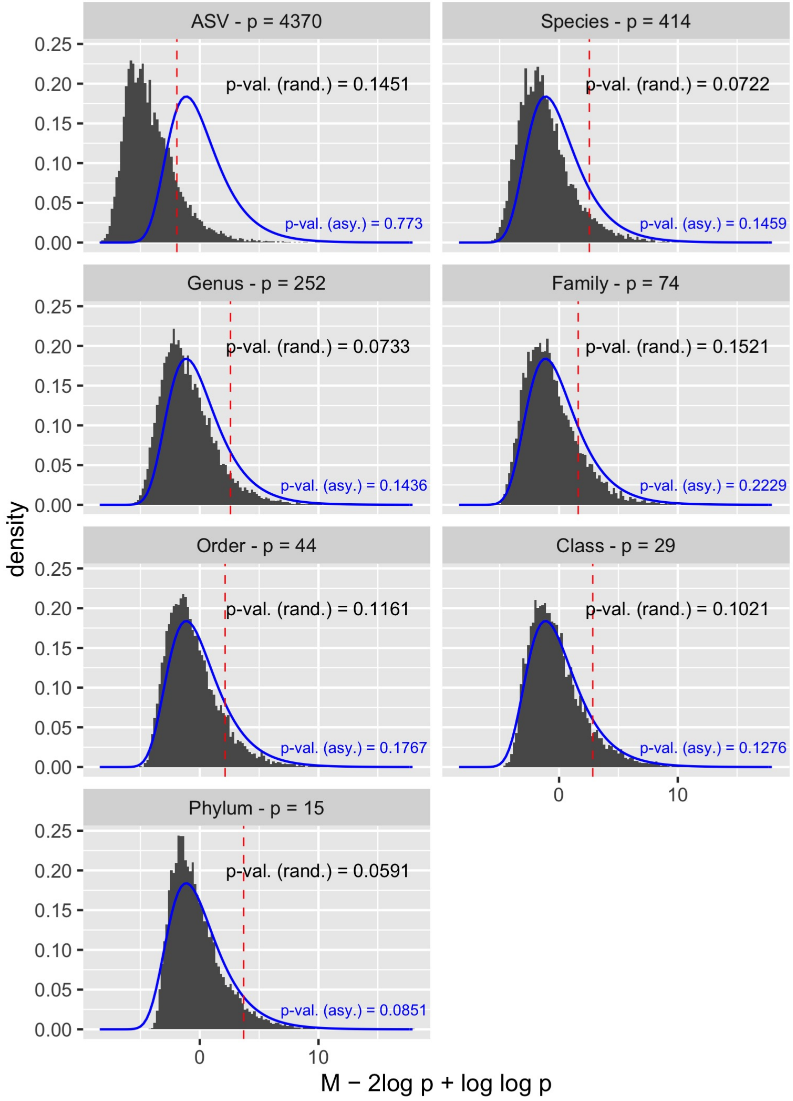

Fig J. Permutation-based (grey) and asymptotic (blue) null randomization distributions for the air pollution reduction hypothetical experiment.

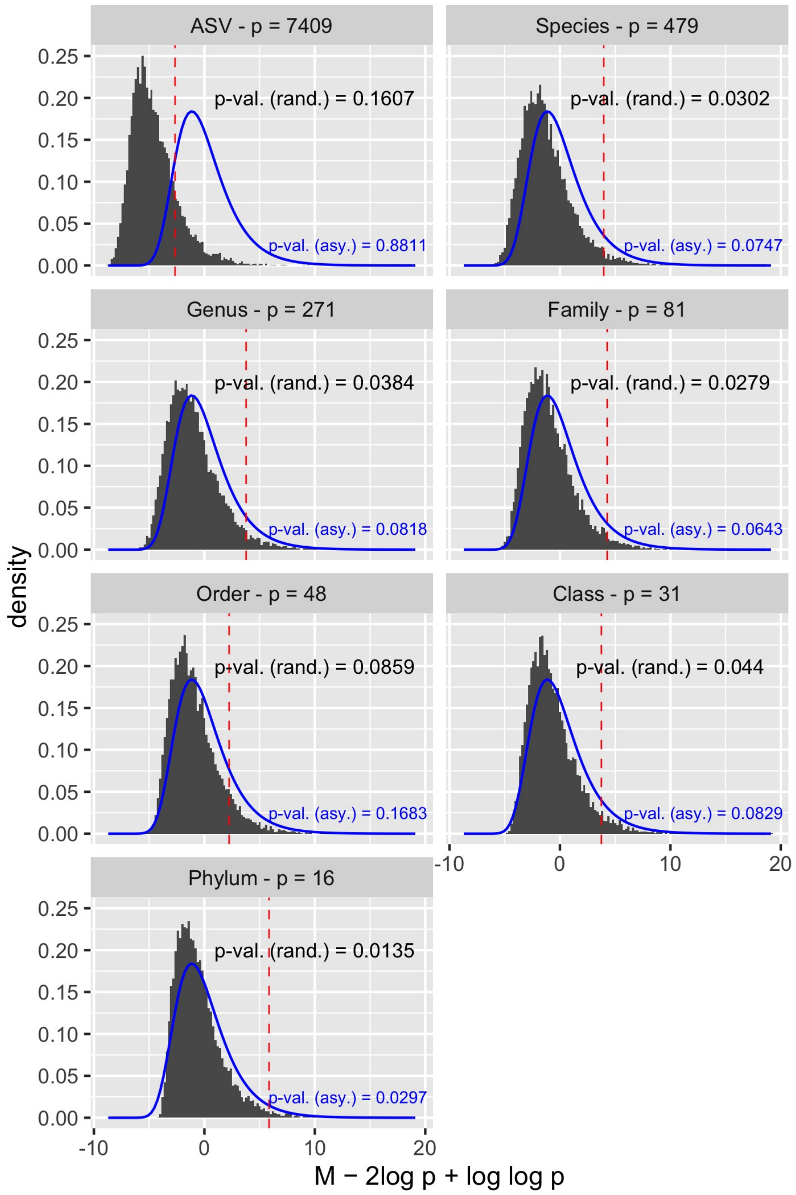

Fig K. Permutation-based (grey) and asymptotic (blue) null randomization distributions for the smoking prevention hypothetical experiment.

Reference selection for DACOMP

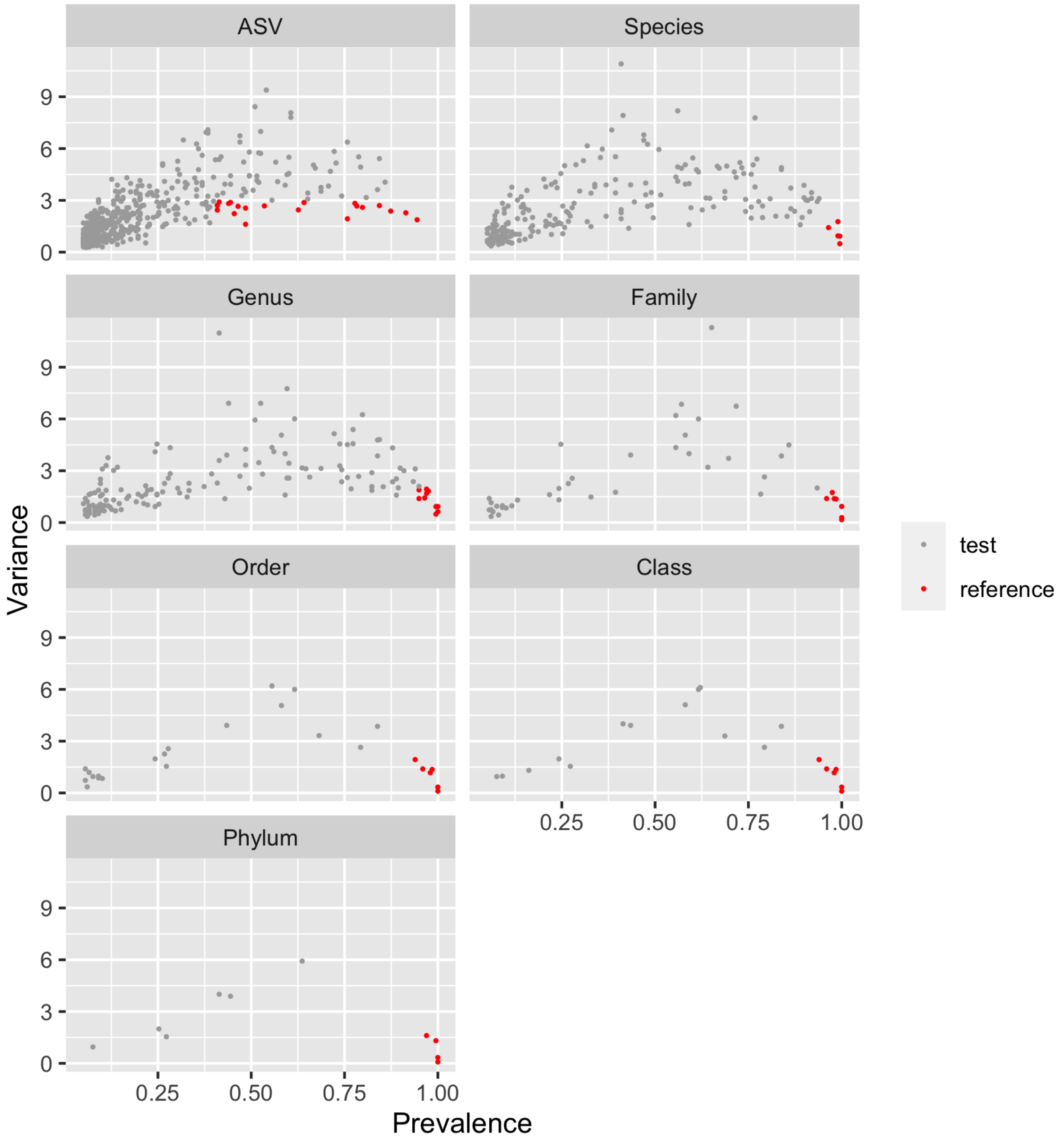

Fig L. Reference set selection in the air pollution reduction experiment. A taxa enters the set $R = (r_1, ..., r_F)$ if it has low variance (< 2) and high prevalence ($> 90\%$). For the analyses at the ASV level, we chose the variance to be < 3 and the prevalence to be $> 40\%$ as thresholds in order the have at least one reference per subject.

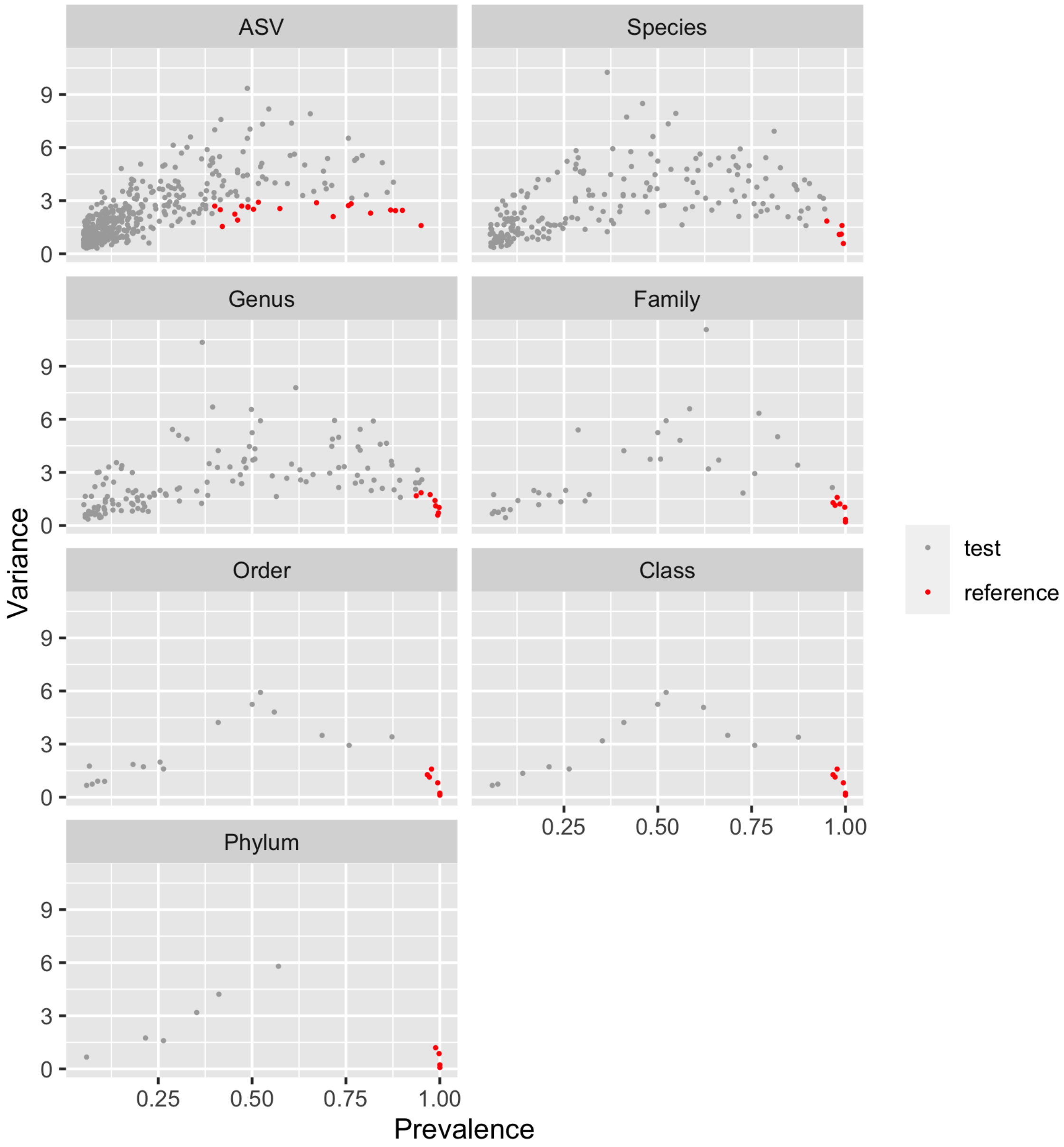

Fig M. Reference set selection in the smoking prevention experiment. A taxa enters the set $R = (r_1, ..., r_F)$ if it has low variance (< 2) and high prevalence ($> 90\%$). For the analyses at the ASV level, we chose the variance to be < 3 and the prevalence to be $> 40\%$ as thresholds in order the have at least one reference per subject.

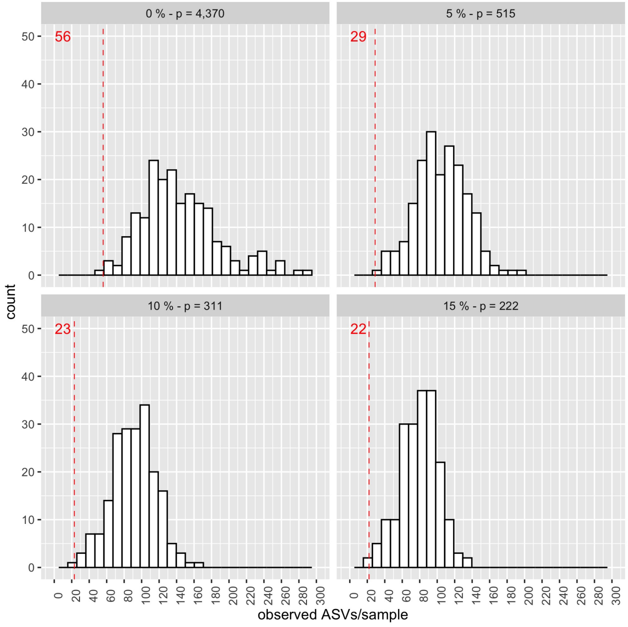

Fig N. Distribution of number of ASVs per sample when data is filtered at different ASV prevalence thresholds (0%, 5%, 10%, 15%) in the air pollution reduction experiment. Red value: minimum observed ASVs per sample.

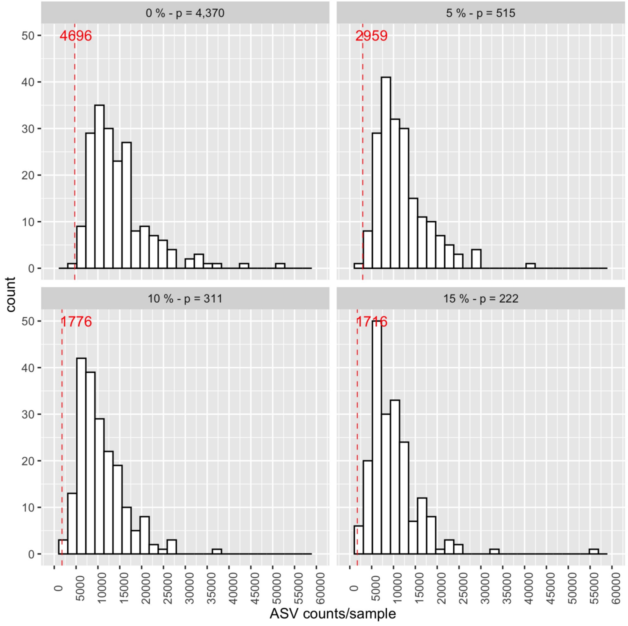

Fig O. Distribution of the total ASV counts per sample when data is filtered at different ASV prevalence thresholds (0%, 5%, 10%, 15%) in the air pollution reduction experiment. Red value: minimum ASV counts per sample.

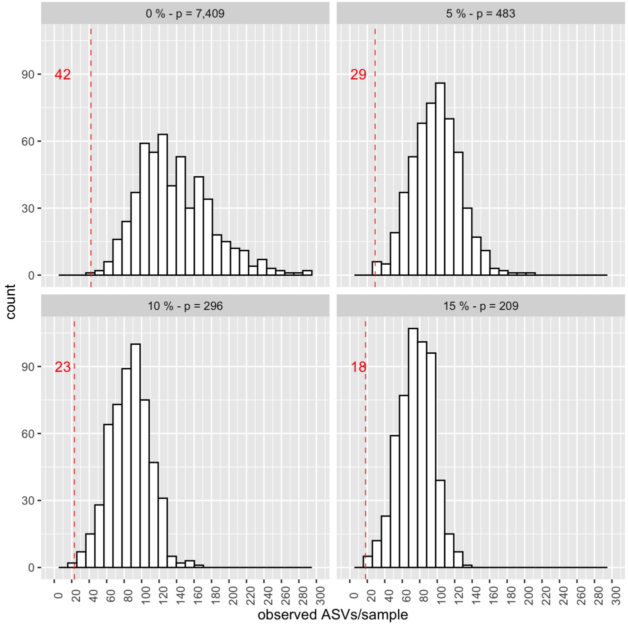

Fig P. Distribution of number of ASVs per sample when data is filtered at different ASV prevalence thresholds (0%, 5%, 10%, 15%) in the smoking prevention reduction experiment. Red value: minimum observed ASVs per sample.

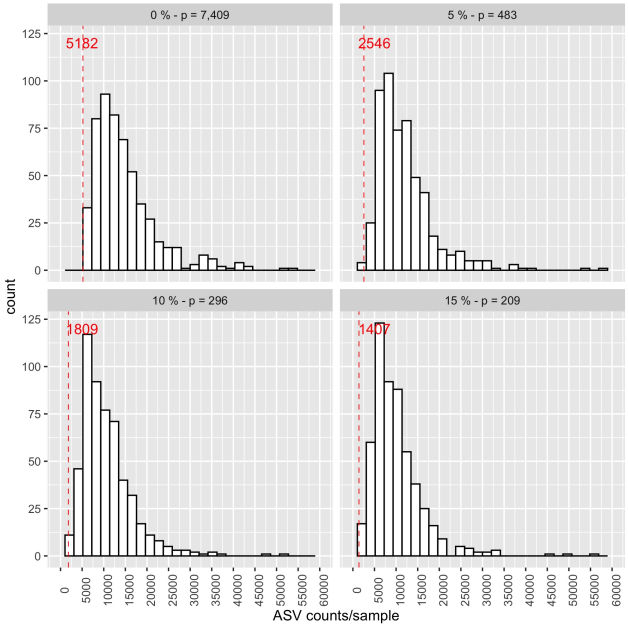

Fig Q. Distribution of the total ASV counts per sample when data is filtered at different ASV prevalence thresholds (0%, 5%, 10%, 15%) in the smoking prevention experiment. Red value: minimum ASV counts per sample.

	Kingdom	Phylum	Class	Order	Family	Genus	Species	p-value$_{adj}$
**ASV**	Bacteria	Firmicutes	Clostridia	Clostridiales	Ruminococcaceae	Anaerotruncus	NA	0.1461 (+)
p = 515	Bacteria	Firmicutes	Clostridia	Clostridiales	Ruminococcaceae	Anaerotruncus	NA	0.0362 (-)
**Species**	Bacteria	Firmicutes	Clostridia	Clostridiales	Lachnospiraceae	Blautia	faecis	0.0357 (+)
p = 220	Bacteria	Firmicutes	Clostridia	Clostridiales	Lachnospiraceae	Marvinbryantia	NA	0.0181 (+)
**Genus** p = 149	Bacteria	Firmicutes	Clostridia	Clostridiales	Lachnospiraceae	Marvinbryantia	NA	0.0120 (+)
**Family** p = 44								
**Order** p = 25								
**Class** p = 19								
**Phylum** p = 10								

Table B. Air pollutiion reduction experiment results. Differentially abundant taxa and adjusted Fisher p-values for 10,000 iterations at 5% prevalence filtering. Selected adjusted p-values ≤ 0.2 (sign of abundance difference: y(1) - y(0)).

	Kingdom	Phylum	Class	Order	Family	Genus	Species	p-value$_{adj}$
**ASV** p = 483	Bacteria	Firmicutes	Clostridia	Clostridiales	Ruminococcaceae	Ruminococcus-1	NA	0.1250 (+)
	Bacteria	Firmicutes	Clostridia	Clostridiales	Ruminococcaceae	Ruminococcaceae-UCG-002	NA	0.1458 (+)
	Bacteria	Firmicutes	Clostridia	Clostridiales	Lachnospiraceae	Lachnospiraceae-NK4A136-group	NA	0.1124 (+)
**Species**	Bacteria	Firmicutes	Clostridia	Clostridiales	Christensenellaceae	Christensenellaceae-R-7-group	NA	0.0201 (+)
p = 211	Bacteria	Firmicutes	Clostridia	Clostridiales	Ruminococcaceae	Ruminococcaceae-UCG-005	NA	0.1124 (+)
	Bacteria	Firmicutes	Clostridia	Clostridiales	Ruminococcaceae	Ruminococcaceae-UCG-003	NA	0.1297 (+)
	Bacteria	Firmicutes	Clostridia	Clostridiales	Lachnospiraceae	Coprococcus-1	catus	0.0392 (+)
	Bacteria	Tenericutes	Mollicutes	NB1-n	NA	NA	NA	0.1458 (+)
	Bacteria	Tenericutes	Mollicutes	Mollicutes-RF9	NA	NA	NA	0.1791 (+)
	Bacteria	Firmicutes	Clostridia	Clostridiales	Ruminococcaceae	Ruminococcaceae-UCG-002	NA	0.1476 (+)
	Bacteria	Firmicutes	Clostridia	Clostridiales	Ruminococcaceae	Ruminococcaceae-UCG-003	NA	0.0127 (+)
	Bacteria	Firmicutes	Clostridia	Clostridiales	Ruminococcaceae	Ruminococcaceae-UCG-005	NA	0.1975 (+)
	Bacteria	Firmicutes	Clostridia	Clostridiales	Ruminococcaceae	Ruminococcus-1	NA	0.1691 (+)
**Genus**	Bacteria	Firmicutes	Clostridia	Clostridiales	Ruminococcaceae	Ruminococcaceae-NK4A214-group	NA	0.1476 (+)
p = 140	Bacteria	Firmicutes	Clostridia	Clostridiales	Christensenellaceae	Christensenellaceae-R-7-group	NA	0.0611 (+)
	Bacteria	Firmicutes	Clostridia	Clostridiales	Lachnospiraceae	Lachnospira	NA	0.0377 (+)
	Bacteria	Firmicutes	Clostridia	Clostridiales	Lachnospiraceae	Lachnospiraceae-NK4A136-group	NA	0.1781 (+)
	Bacteria	Firmicutes	Clostridia	Clostridiales	Lachnospiraceae	Coprococcus-1	NA	0.0611 (+)
	Bacteria	Tenericutes	Mollicutes	NB1-n	NA	NA	NA	0.1882 (+)
	Bacteria	Tenericutes	Mollicutes	Mollicutes-RF9	NA	NA	NA	0.1166 (+)
	Bacteria	Firmicutes	Clostridia	Clostridiales	Christensenellaceae	NA	NA	0.0199 (+)
**Family**	Bacteria	Tenericutes	Mollicutes	NB1-n	NA	NA	NA	0.0450 (+)
p = 41	Bacteria	Tenericutes	Mollicutes	Mollicutes-RF9	NA	NA	NA	0.0512 (+)
**Order**	Bacteria	Tenericutes	Mollicutes	NB1-n	NA	NA	NA	0.0375 (+)
p = 22	Bacteria	Tenericutes	Mollicutes	Mollicutes-RF9	NA	NA	NA	0.0404 (+)
**Class** p = 19	Bacteria	Tenericutes	Mollicutes	NA	NA	NA	NA	0.0039 (+)
**Phylum** p = 10	Bacteria	Tenericutes	NA	NA	NA	NA	NA	0.0018 (+)

Table C. Smoking prevention experiment results. Differentially abundant taxa and adjusted Fisher p-values for 10,000 iterations at 5% prevalence filtering. Selected adjusted p-values ≤ 0.2 (sign of abundance difference: y(1) - y(0)).

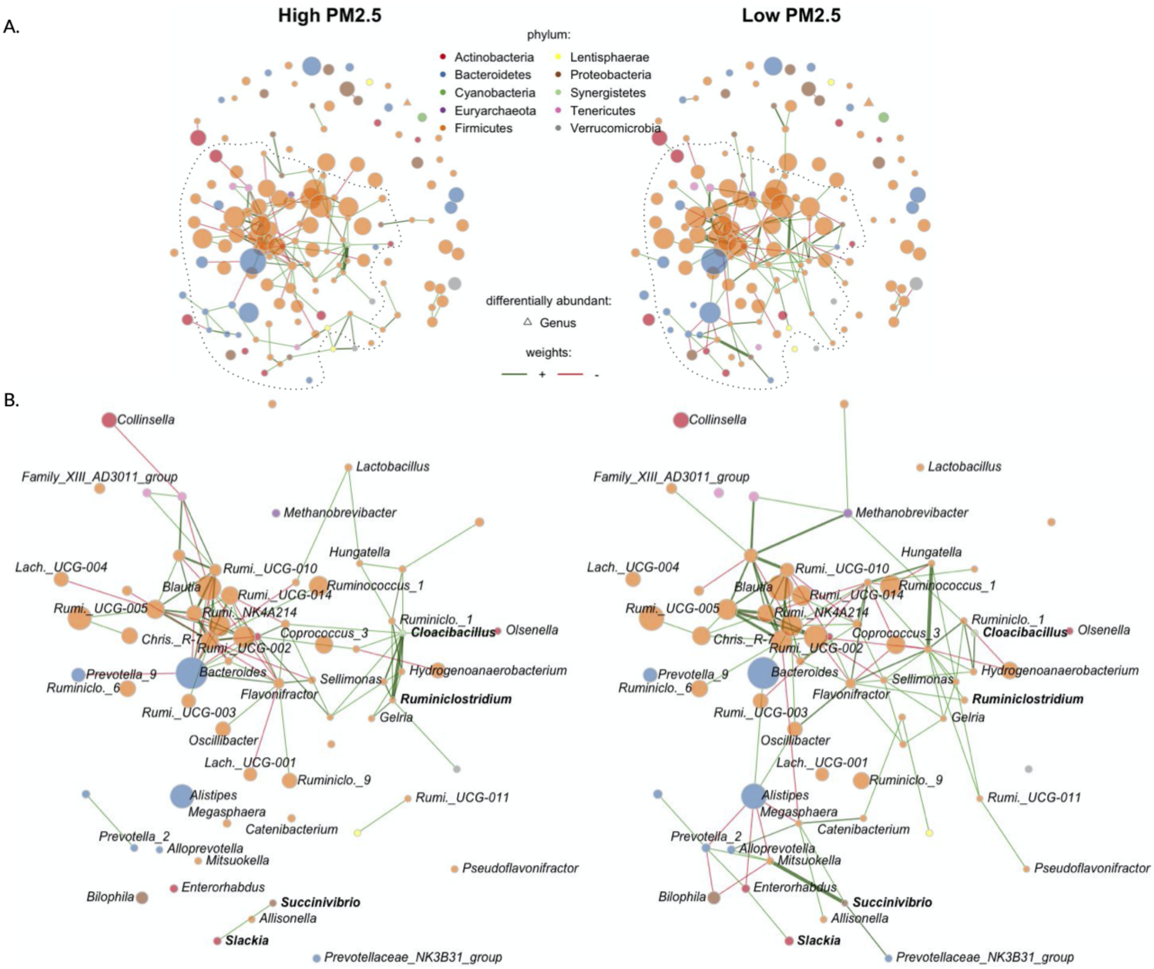

Fig R. Genus-genus associations for subject under the air pollution reduction experiment vs. not (n = 99, p = 149). (A) Visualization of the between genera partial correlations estimated with the SPIEC-EASI method. Edges thickness is proportional to partial correlation, and color to direction: red: negative partial correlation, green: positive partial correlation. Node size is proportional to the centered log ratio of the genus abundances, and color is according to phyla. Triangle shaped nodes are differentially abundant (see Figure 3). (B) Zoom in largest connected component and differential associations (bold genera).

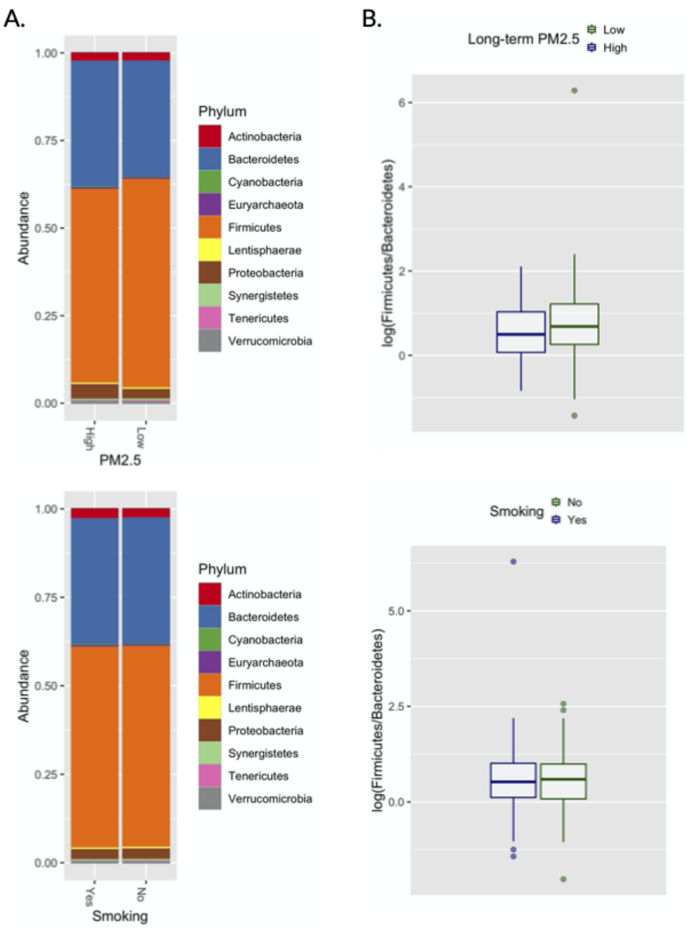

Fig S. Phyla comparison.

Sensitivity analysis

		Air pollution ($PM_{2.5}$)				Smoking			
		$\geq 13.0\ \mu g/m^3$ $n = 158$		$\leq 10.3\ \mu g/m^3$ $n = 158$		Smoker $n = 290$		Never-Smoker $n = 290$	
		Mean	St. d.	Mean	St. d.	Mean	St. d.	Mean	St. d.
Age		60.0	12.8	59.6	12.6	54.6	9.4	54.8	11.4
Body Mass Index		28.1	5.6	28.1	5.5	27.0	4.9	27.0	4.5
Alcohol intake (g/day)		15.3	17.2	16.4	20.1	15.1	19.9	14.6	18.0
Years of education		11.8	2.8	11.4	2.5	11.7	2.4	12.0	2.6
		N	%	N	%	N	%	N	%
Sex	F	75	23.7	73	23.1	142	24.5	145	25.0
	M	83	26.3	85	26.9	148	25.5	145	25.0
Smoking	Ex-S.	60	19.0	57	18.0	-	-	-	-
	Never-S.	83	26.3	83	26.3	-	-	-	-
	Smoker	15	4.7	18	5.7	-	-	-	-
Diabetes	No	142	44.9	146	46.2	272	46.9	268	46.2
	Yes	16	5.1	12	3.8	18	3.1	22	3.8
Phys. Activity	No	70	22.2	66	20.9	137	23.6	127	21.9
	Yes	88	27.8	92	29.1	153	26.4	163	28.1

Table D. Sensitivity analysis - Baseline characteristics of the study population in the air pollution reduction (left table) and smoking prevention experiments (right table). Continuous variables: mean and standard deviation (St. d.). Categorical variables: number of samples per category (N) and proportion of category (%).

Air pollution

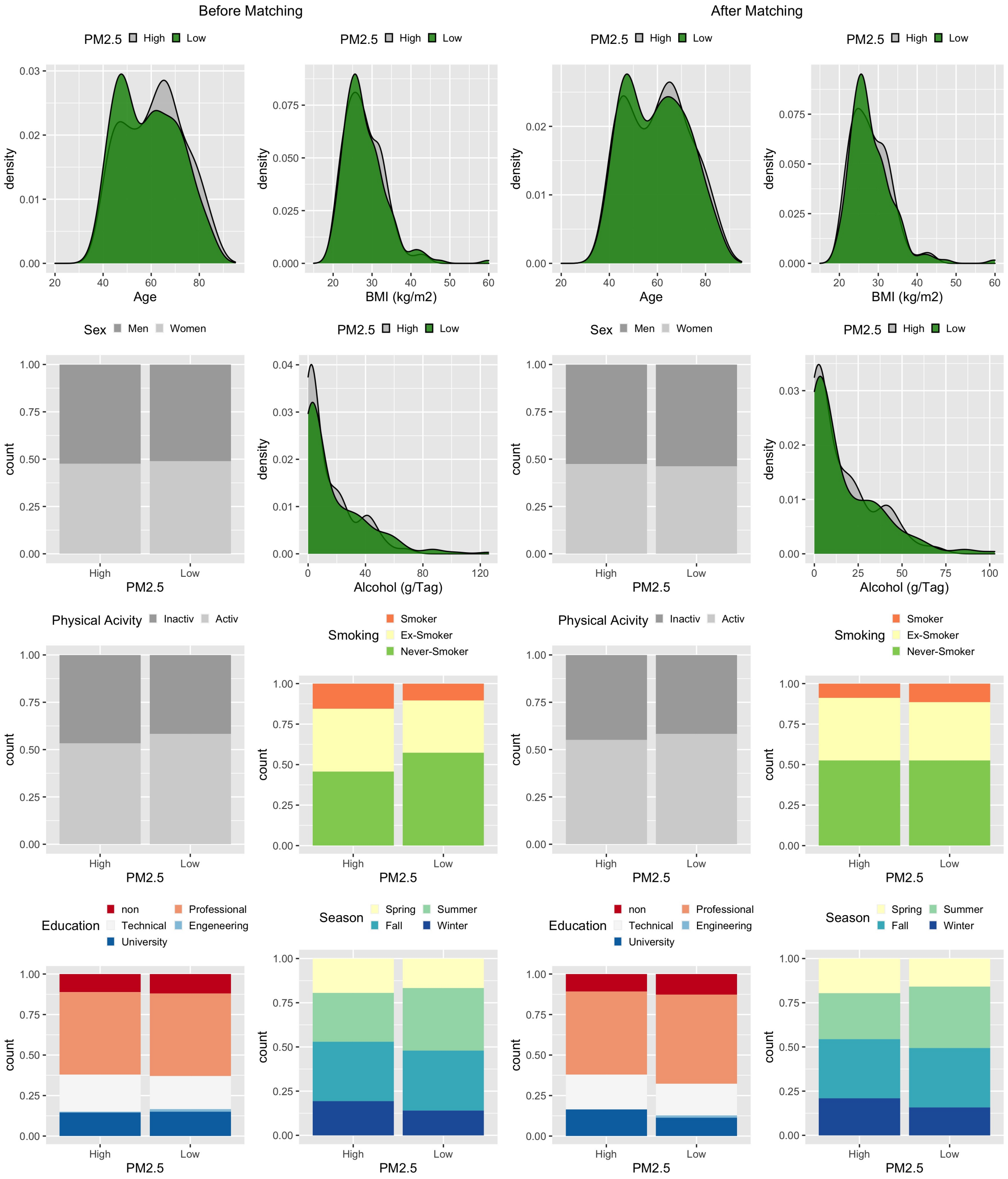

Fig T. Sensitivity analysis - Empirical distributions of the covariates among the subjects under the intervention vs. not in the original (left panel) and the balanced (right panel) data for the air pollution reduction hypothetical experiment.

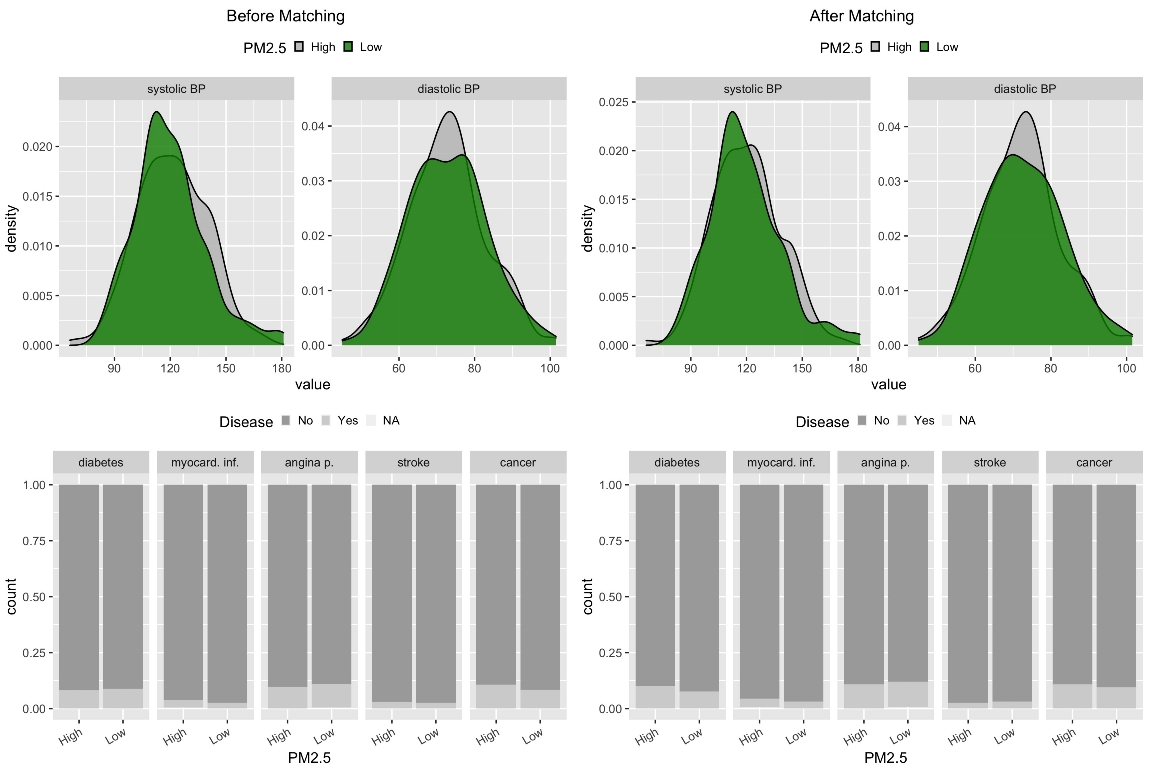

Fig U. Sensitivity analysis - Empirical distributions of the diseases covariates among the subjects under the intervention vs. not in the original (left panel) and the balanced (right panel) data for the air pollution reduction hypothetical experiment.

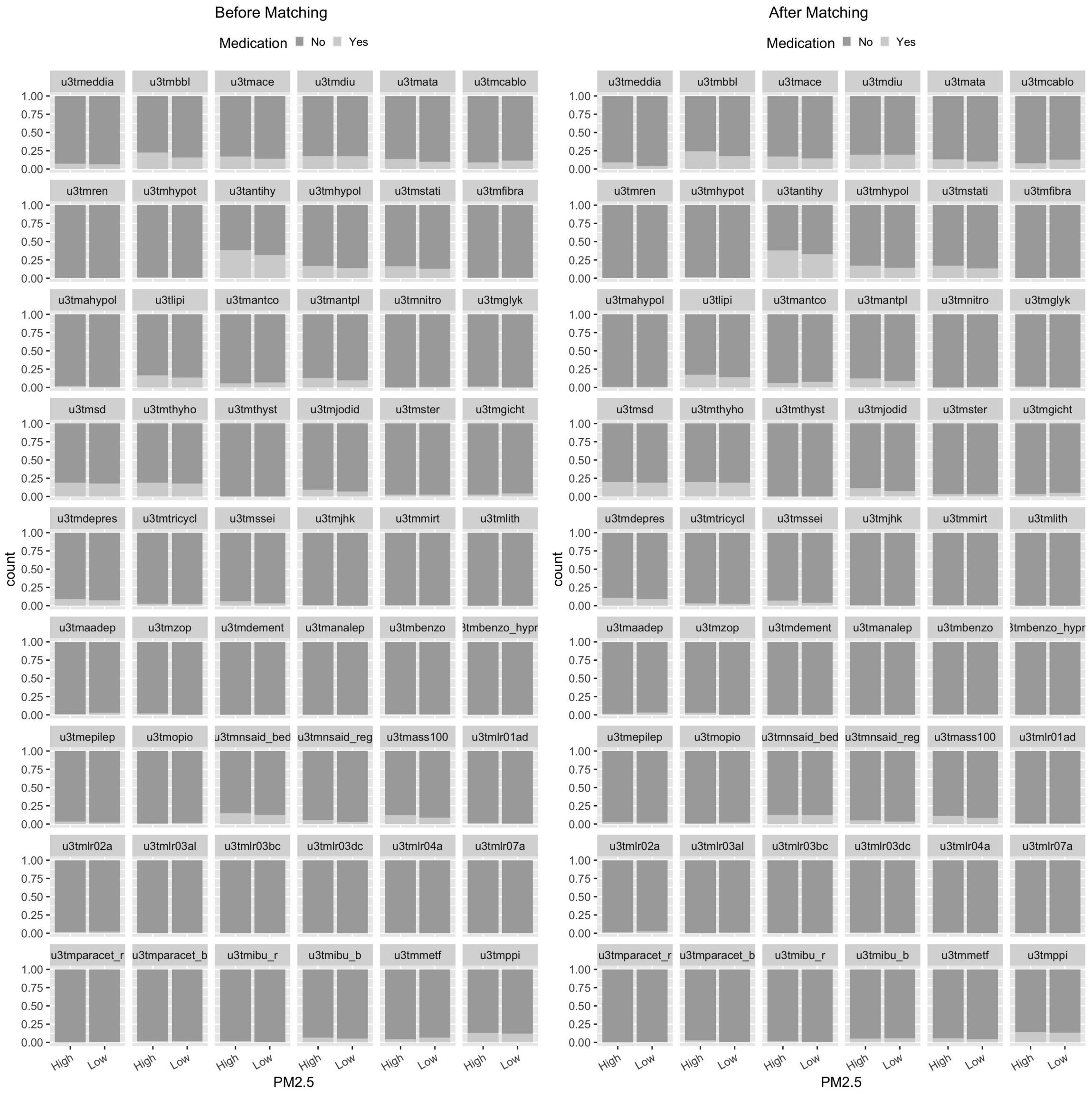

Fig V. Sensitivity analysis - Empirical distributions of the medication covariates among the subjects under the intervention vs. not in the original (left panel) and the balanced (right panel) data for the air pollution reduction hypothetical experiment.

Smoking

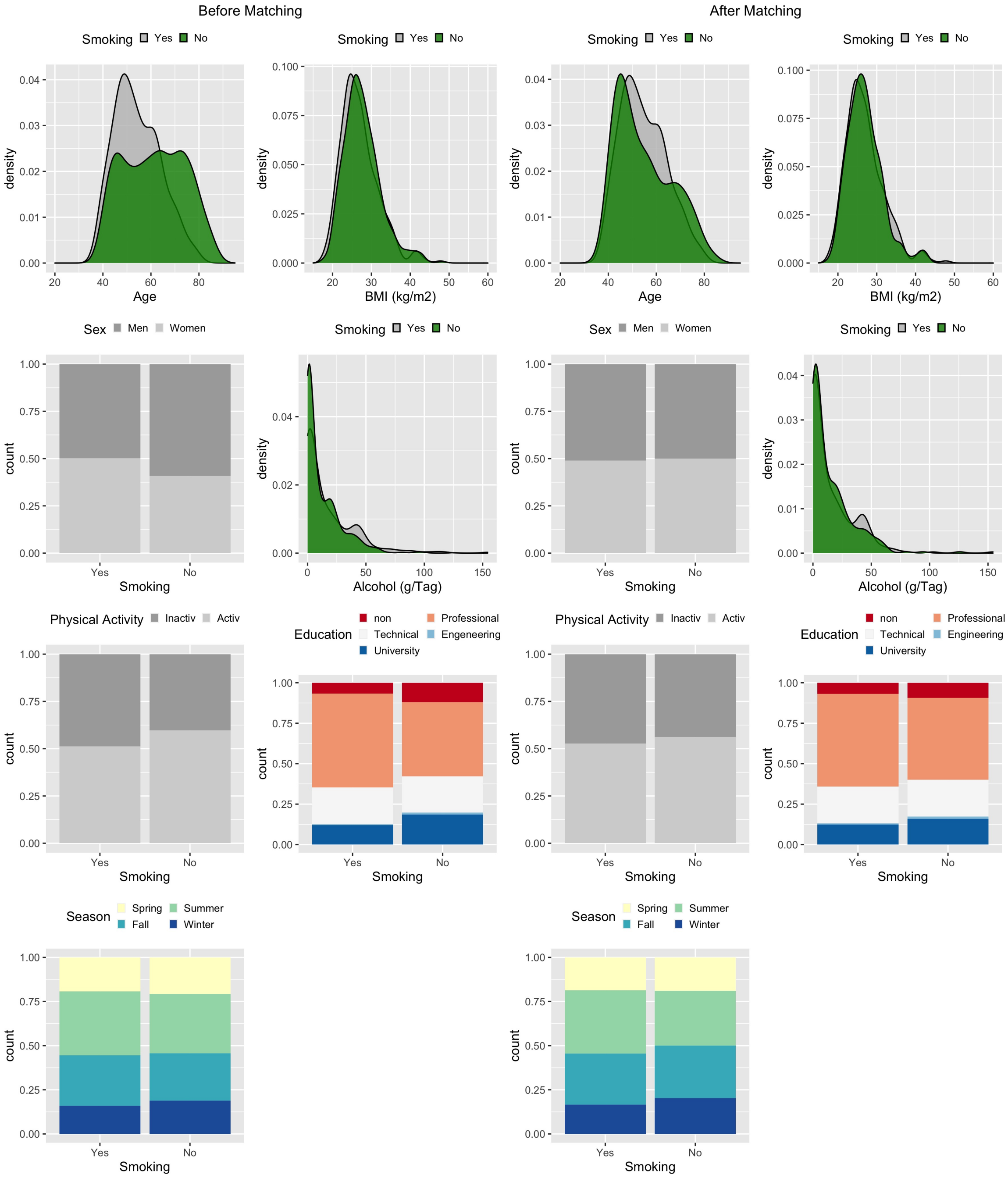

Fig W. Sensitivity analysis - Empirical distributions of the covariates among the subjects under the intervention vs. not in the original (left panel) and the balanced (right panel) data for the smoking prevention hypothetical experiment.

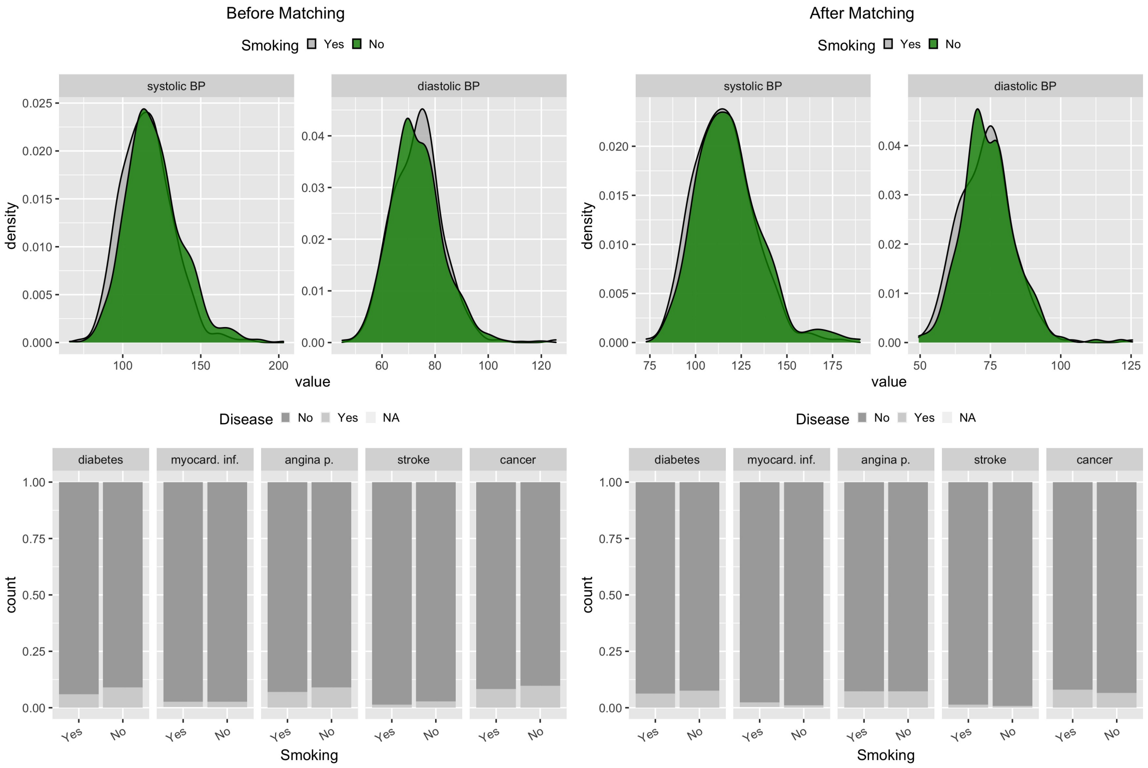

Fig X. Sensitivity analysis - Empirical distributions of the diseases covariates among the subjects under the intervention vs. not in the original (left panel) and the balanced (right panel) data for the smoking prevention hypothetical experiment.

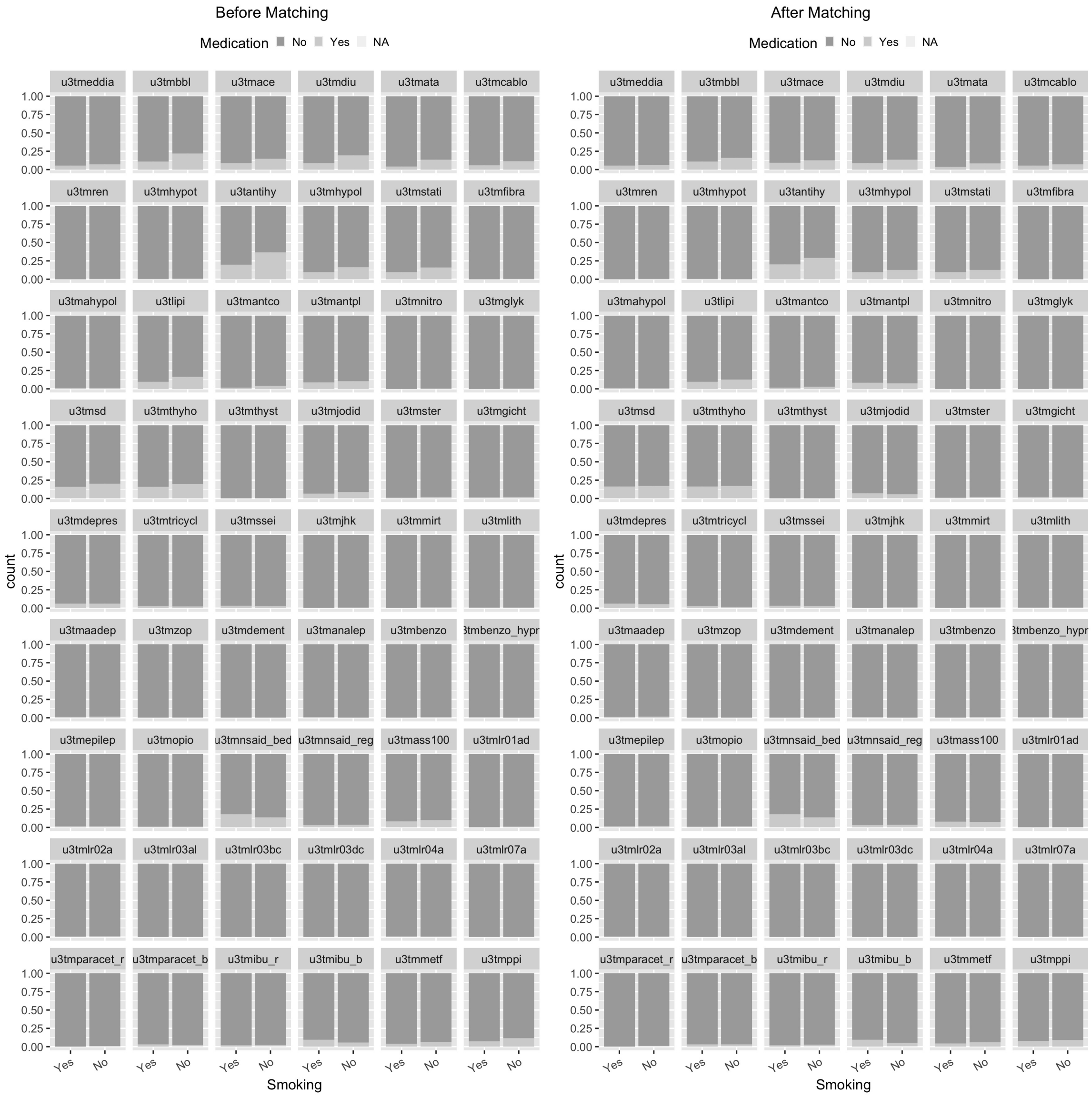

Fig Y. Sensitivity analysis - Empirical distributions of the medication covariates among the subjects under the intervention vs. not in the original (left panel) and the balanced (right panel) data for the smoking prevention hypothetical experiment.

Results

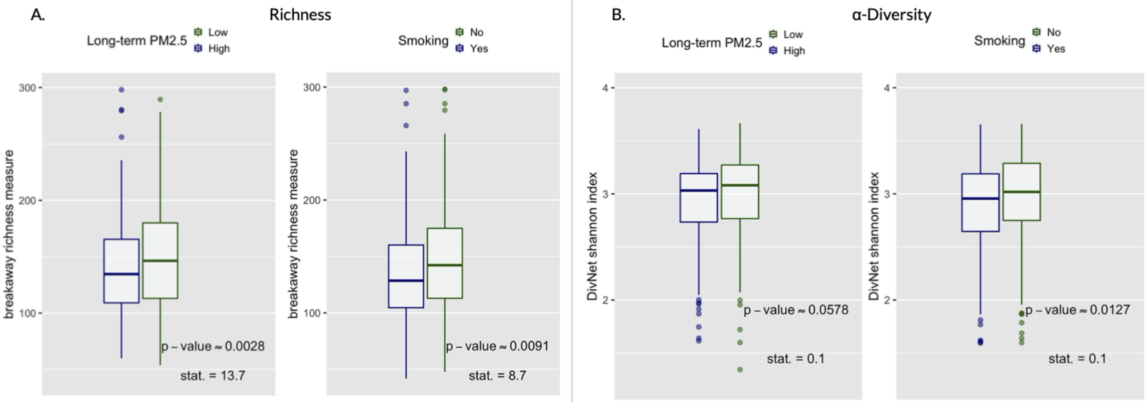

Fig Z. Sensitivity analysis - Richness and α-diversity. Boxplots (with median), values of the test-statistics from the `betta` regression, and one-sided randomization-based p-values for 10,000 permutations of the intervention assignment following a matched-pair design.

	**Air pollution**			**Smoking**		
distance	test-statistic	p-value	p-value$_{adj}$	test-statistic	p-value	p-value$_{adj}$
UniFrac	15.1	0.1950	0.3984	91.0	0.0004	0.0010
Aitchison	123180.7	0.2361	0.4658	432662.8	0.0003	0.0003
Jaccard	29.1	0.2238	0.4467	104.4	0.0001	0.0003
Gower	0.1	0.0223	0.0596	0.2	0.0046	0.0132

Table E. Sensitivity analysis - β-diversity. Microbiome Regression-based Kernel Association Test (`MiRKAT`), unadjusted and adjusted one-sided randomization-based p-values for 10,000 permutations of the intervention assignment following a matched-pair design.

		ASV	Species	Genus	Family	Order	Class	Phylum
**Air Pollution**	nb. of taxa (p)	5,635	595	329	100	59	36	18
	test statistic	13.5	8.3	9.2	8.2	7.2	5.6	5.3
	p-value	0.1070	0.5938	0.3017	0.1839	0.2046	0.3602	0.2429
**Smoking**	nb. of taxa (p)	7,793	595	278	84	51	31	15
	test statistic	19.5	23.8	19.0	14.2	12.7	13.5	14.3
	p-value	0.0048	0.0004	0.0019	0.0094	0.0108	0.0061	0.0016

Table F. Sensitivity analysis - Compositional equivalence test. Test statistic for high-dimensional data and one-sided randomization-based p-values for 10,000 permutations of the intervention assignment following a matched-pair design.

	Kingdom	Phylum	Class	Order	Family	Genus	Species	p-value$_{adj}$
	Bacteria	Firmicutes	Clostridia	Clostridiales	Ruminococcaceae	Ruminococcaceae-UCG-002	NA	0.0129 (+)
	Bacteria	Firmicutes	Clostridia	Clostridiales	Ruminococcaceae	Ruminococcaceae-UCG-003	NA	0.0129 (+)
	Bacteria	Firmicutes	Clostridia	Clostridiales	Ruminococcaceae	Ruminococcaceae-UCG-005	NA	0.0252 (+)
	Bacteria	Firmicutes	Clostridia	Clostridiales	Ruminococcaceae	Ruminococcaceae-UCG-010	NA	0.0949 (+)
**Genus**	Bacteria	Firmicutes	Clostridia	Clostridiales	Ruminococcaceae	Ruminococcus-1	NA	0.0725 (+)
p = 142	Bacteria	Firmicutes	Clostridia	Clostridiales	Ruminococcaceae	Ruminococcaceae-NK4A214-group	NA	0.0376 (+)
	Bacteria	Firmicutes	Clostridia	Clostridiales	Christensenellaceae	Christensenellaceae-R-7-group	NA	0.0376 (+)
	Bacteria	Firmicutes	Clostridia	Clostridiales	Lachnospiraceae	Lachnospira	NA	0.0129 (+)
	Bacteria	Firmicutes	Clostridia	Clostridiales	Lachnospiraceae	Lachnospiraceae-UCG-001	NA	0.0376 (+)
	Bacteria	Firmicutes	Clostridia	Clostridiales	Lachnospiraceae	Lachnospiraceae-UCG-010	NA	0.1884 (+)
	Bacteria	Firmicutes	Clostridia	Clostridiales	Lachnospiraceae	Lachnospiraceae-NK4A136-group	NA	0.0376 (+)
	Bacteria	Tenericutes	Mollicutes	NB1-n	NA	NA	NA	0.0129 (+)

Table G. Sensitivity analysis - Smoking prevention experiment results. Differentially abundant taxa and adjusted Fisher p-values for 10,000 iterations at 5% prevalence filtering. Selected adjusted p-values ≤ 0.2 (sign of abundance difference: y(1) - y(0)).
