# Supplementary material for: A randomization-based causal inference framework for uncovering environmental exposure effects on human gut microbiota
Source: PLoS Comput Biol. 2022 May 9;18(5):e1010044. doi: 10.1371/journal.pcbi.1010044 (PMC9129050; doi:10.1371/journal.pcbi.1010044)
Supplement: Attachment — Submitted filename: PLOS_CB_report.pdf [file pcbi.1010044.s002.pdf]

Referee report on "A randomization-based causal inference framework for uncovering environmental exposure effects on human gut microbiota"

This paper describes a matching-based causal inference framework for the microbiome data. The proposed method uses the dataset generetad from an observational study to create a pseudo-randomized experiment, based on which the estimates and testing results are endowed with causal interpretation. The paper is well written and the central task, causal inference of microbiome data, is important to the field. There are, however, several points if addressed would make the paper stronger.

- **Matching algorithm**. The proposed matching algorithm is purely based on the list of covariates that are observed in the study. This is viable when the number of covariates is relatively small but will reduce the number of matched pairs significantly otherwise. In the data example of this manuscript, we can already see the effect of matching on covariates: the original 1,967 subjects were reduced to fewer than 600 subjects after matching. It would be interesting to compare the proposed matching algorithm with the more commonly used propensity score matching (Rosenbaum and Rubin, 1983). Propensity score matching (PSM) takes care of the potential problems associated with high dimensionality of the covariate space and can yield more matched pairs and lead to better power. The tuning process is more straightforward since there's only one tuning paremeter in PSM (i.e., the maximal gap in propensity score)

(Wu et al., 2018; Ren et al., 2021).

- **Tuning approach for the matching algorithm**. The manuscript only listed the selected δ_k but didn't explain how they were derived. Referring to the diagnostic plots is not sufficient. The authors need to provide more details on how they arrive at these δ_k, especially how they define the metric of covariate balance and whether δ_k is selected by minimizing this metric.

- **Propogation of uncertainty**. Although the authors acknowledged that exact alpha- and beta-diversities cannot be recovered from the ASV data and made effort to use statistical models to estimate both diversities, whether the uncertainty of these estimates is propogated into the testing procedure is still of question. I noticed that the authors used standard errors of the estimates as a covariate in the hierarchical regression models, but I'm not convinced this is sufficient for uncertainty propogation. It would be useful to consider non-parameteric approaches, such as bootstrap, to address this issue. A potential bootstrap approach could be to first generate bootstrapped data and then carry out the permutations.

- **Lack of simulation analysis**. The authors proposed a causal inference approach for microbiome data and used it to analysis real data without providing any evidence whether the approach can indeed recovers the underlying causal relationships. A simulation study is critical to justify the causal interpretation of the proposed estimates and tests. Simulating microbiome data requires more effort so that the common properites, such as compositionality and zero-inflation, can be preserved. The authors can use resampling approaches (McMurdie and Holmes, 2013) or training a generative model using real data (Ma et al., 2021).

- **Minor comments.**

  1. In captions of Fig. 2 and Tables 2-3, the authors suggested one-sided tests were

used. However, in the description of the testing procedure in pg. 18, the p-values seem to be calculated with a two-sided alternative.

2. What might be the reasons for the changes in statistical signficance when the data are aggregated into different phylogenic levels for the tests of compositional mean differences? I would imagine that if lower levels (e.g. genus) have strong significance, so does the high levels (e.g. faimly). This is not the case in the contrast between high vs. low PM exposures.

3. A similar question is also for the results of beta-diversity. Why Aitchinson and Jaccard have much larger p-values than the other two beta-diversity for the contrast between high vs. low PM exposures?

4. The authors suggested that the permutations have to perserve the pairing structure (e.g. pg 21, line -4). How is this achieved? I guess it's by permuting the case/control status within each pair?

5. The multiple testing correction procedue is not well described. For example, the role of raw p-values of the observed statistics is not clear and how do you get the unadjusted p-values for each $T_{\beta}^{h,iter}$?

6. How do you handle zeroes in C_i^a when calculating L_i^a?

7. Typos.

   - pg. 16, line -5, "guaranty" should be "gaurantee".
   - pg. 19, line -15, "$H_{0,B}$" should be "$H_{0,R}$".
   - pg. 20. "In our case a hypothetical ..." is not a complete sentence.
   - pg. 20. In the definition of $g(C_i)$, $1/a$ should be $1/A$.

References

Ma, S., B. Ren, H. Mallick, Y. S. Moon, E. Schwager, S. Maharjan, T. L. Tickle, Y. Lu, R. N. Carmody, E. A. Franzosa, et al. (2021). A statistical model for describing and

simulating microbial community profiles. *bioRxiv*.

McMurdie, P. J. and S. Holmes (2013). phyloseq: an r package for reproducible interactive analysis and graphics of microbiome census data. *PloS one 8*(4), e61217.

Ren, B., X. Wu, D. Braun, N. Pillai, and F. Dominici (2021). Bayesian modeling for exposure response curve via gaussian processes: Causal effects of exposure to air pollution on health outcomes. *arXiv preprint arXiv:2105.03454*.

Rosenbaum, P. R. and D. B. Rubin (1983). The central role of the propensity score in observational studies for causal effects. *Biometrika 70*(1), 41–55.

Wu, X., F. Mealli, M.-A. Kioumourtzoglou, F. Dominici, and D. Braun (2018). Matching on generalized propensity scores with continuous exposures. *arXiv preprint arXiv:1812.06575*.
