# Supplementary material for: A randomization-based causal inference framework for uncovering environmental exposure effects on human gut microbiota
Source: PLoS Comput Biol. 2022 May 9;18(5):e1010044. doi: 10.1371/journal.pcbi.1010044 (PMC9129050; doi:10.1371/journal.pcbi.1010044)
Supplement: Attachment — Submitted filename: review.pdf [file pcbi.1010044.s003.pdf]

Review of: "A randomization-based causal inference framework for uncovering environmental exposure effects on human gut microbiota"

by Sommer et al.

The paper focuses on the analysis of a microbiome data set from the KORA cohort study. The goal was to consider the question of whether or not (a) air pollution reduction and (b) smoking cessation changed the microbiome. While the standard microbiome analysis is performed on the data sets, the strength of the paper lies in applying the **Rubin Causal Model** to the data sets to go beyond correlational and associational explorations.

This is done using the carefully laid out 4-step process consisting of conceptual, design, analysis and summary. The statistical work is done well, except for the language about p-values and accepting or rejecting the null hypotheses.

The organization of the paper is awkward. The causal analysis is confined to the discussion section. All of this should belong to the Methods section, as done here. But the results are not discussed in the Results section, and no discussion is provided.

Overall, this is a good paper with solid analyses and deserves to be published.

Major Comments

1. I understand the instinct to follow the ASA suggestion to avoid the arbitrary p-value threshold of 0.05. However, it does not eliminate the arbitrariness from their decisions to decide when a p-value is "small" or "low", and therefore, significant. On p4, the authors claim to "only describe the Fisherian p-values", but the paper does accept or reject several null hypotheses with no uniformity or logic to dictate those action(s). For example, Fig. 2 suggests rejecting the null hypotheses of no difference in richness and diversity due to air pollution, but the values for smoking are hardly small (about 0.15), and yet they are being rejected. When is it "small" and who decides and on what basis? Additionally, I suspect that there are threshold built into tools like SPIEC-EASI, aong others that they have employed. A statement on p13 states that they only reject null hypotheses and that it indicates further study and not that they are different. I find this logic strange. With no explicit threshold, the explanations seems murky and arbitrary. Arbitrarily calling for "further scrutiny" is very unsatisfying. Can the authors provide an "algorithm" for when they reject and when they call for further study?

2. Shouldn't there be a discussion on the anomaly of alpha and beta diversity giving two (marginally) different messages?

3. The "Differential genus-genus associations" were a bust. The p-values were not really small, but two of the edges were arbitrarily called out as significant.

4. I have suggested many of these statements about rejecting the null hypotheses to be tempered. These suggestions should also appropriately modify the discussion as well, where these statements have been repeated.

Minor Comments

p1, Abstract In sentence 2, move the word "however" to the start of the sentence.

p6, line 3 I suggest the statement be modified to say something to the effect that "The Fisherian p-values for rejecting the null hypotheses for the smoking prevention data is not strong, and other measures will be explored."

p6, Between-subject variation Same problem as above. This time the roles are switched. And I suggest taking a similar strategy and making a similar statement as above, but about beta diversity with air pollution.

p6, Between-subject variation Delete the phrase "p-values are small" or else provide a clear logic for why.

p7 Rewrite the phrase "p-values are low" as "p-values are lower at the ASV level than at the higher levels of the taxonomy".

p7, 7th line from bottom Delete "do not". I believe you want to reject here for the 11 genera.

p8, 1st line Replace "high-level" by "deeper" or "greater".

p10 I want to see a clear statement that states that the evidence for rejecting the null hypothesis was very marginal, but that aong the edges the ones for which the greatest support was availale were the ones they mention.

p11, Discussion Delete the phrase "Due to the interdisciplinary (epidemiology, microbiome research, and statistics) nature of this paper".

p12, line 2 Rejecting the sharp null hypothesis for the smoking study has inadequate support.

p12, line 19 replace "depict" by "observe"

p12, line 20 replace "genera than reported by Lee" by "genera that were reported by Lee"

p14, line 4 replace "direction than" by "direction as".

p14, line 4 from bottom replace "libraries are then" by "libraries were then"

p15, line 14 replace "16s" by "16S"

p22, line 8 replace "cautiousness" by "caution"
