# Supplementary material for: A randomization-based causal inference framework for uncovering environmental exposure effects on human gut microbiota
Source: PLoS Comput Biol. 2022 May 9;18(5):e1010044. doi: 10.1371/journal.pcbi.1010044 (PMC9129050; doi:10.1371/journal.pcbi.1010044)
Supplement: Attachment — Submitted filename: Response_reviewer_comments_Sommer.pdf [file pcbi.1010044.s004.pdf]

We thank the Guest Editor and three reviewers for their helpful comments. We believe our paper has been greatly improved by incorporating the thoughtful feedback provided. In particular, more details on changes can be found below, with our responses recorded in blue.

GUEST EDITOR'S COMMENTS:

We thank the guest editor for their kind assessment of our work.

Reviewer #2, who is overall very positive, wrote that "the strength of the paper lies in applying the Rubin Causal Model to the data sets to go beyond correlational and associational explorations", and this, I suppose, is a fair summary of your paper's main selling point. Nevertheless, the Reviewer considers the "organization of the paper [as] awkward".

We agree, the original paper organization (i.e., 1. Introduction, 2. Results, 3. Discussion, 4. Methods), which is often recommended by interdisciplinary journals, can be perceived as awkward depending on the audience of the paper. We have taken Reviewer #2's suggestion into account and reordered the sections, i.e., 1. Introduction, 2. Methods, 3. Results, 4. Discussion.

In contrast to Reviewer #1, Reviewer #3 seems to doubt that you are really using a causal frame: "the framework is nothing but just matching". I do not know much about causal inference, myself, but I also had this thought when reading your paper: if normal inference of correlation cannot show direction of causality, how can a matching scheme like your overcome that? I suppose that the answer to this question is the core idea of Rubin's model, and hence, maybe you need add a more thorough introduction to Rubin's methodology, accessible to readers not yet familiar with Rubin's work, and make sure that it is well linked to your actually method and explains how your matching-based scheme allows to actually infer causation rather than only correlation.

We clarified on page 7 the usefulness of a design stage:

"The aim of our pair-matching strategy is to achieve balance in background covariates distributions as it is expected, on average, in randomized experiments. This approach attempts to create exchangeable groups as if the exposure was randomly assigned to each participant given measured covariates, to guarantee exposure assignment is not confounded by the measured background covariates. The exposure assignment mechanism determines which units receive which exposure; in other words, which potential outcomes are observed and which are missing (Imbens & Rubin, 2015). The unconfoundedness of the assignment mechanism given covariates is a key assumption of the Rubin Causal Model."

We attempted to make this clearer in the Introduction with the following sentence on page 3:

"The reason for using this four-stage approach is for the transparency of its assumptions when interpreting results."

If this is still unclear, we are happy to clarify in another way.

Reviewer #1 does not seem to share Reviewer #3's and my doubts about how matching can recover causal relations (and this is why I suppose that my doubts are merely due to my lack of understanding of Rubin's work and how you apply it). Nevertheless, that reviewer has several questions on how you justify your selection of a matching scheme, and how it compares to other state-of-the-art approaches. The reviewer also is unconvinced that your method is actually able to recover causal relationships, and suggests to prove this with simulations.

We took the suggestion of Reviewer #1 and Reviewer #3 and have performed a sensitivity analysis comparing the results obtained after our pair-matching strategy to the more commonly used propensity score matching. We have added a new section on page 20 addressing this:

“To assess whether the pair-matching strategy chosen for the design stage could influence the conclusions of this study, we conducted a sensitivity analysis (see Supplementary Sensitivity Analysis section). For that, we implemented the more commonly-used propensity score matching algorithm (Rosenbaum & Rubin, 1983) and obtained matched samples of: 1) 158 participants living in low $PM_{2.5}$ areas and 158 participants living in higher $PM_{2.5}$ areas, and 2) 290 smokers and 290 never smokers (see Supplementary Table 4 and Figures 20-25 for the balance diagnostics). For both hypothetical randomized experiments, using propensity score matching at the design stage results in analyzing more matched samples. The microbial diversity analyses lead to the same conclusion for both experiments despite different design stages. Overall, we also observe small approximate Fisherian p-values after performing the propensity score matching (in the same way we observe small approximate Fisherian p-values with our pair-matching strategy). The test statistics have the same direction and magnitude. For the air pollution reduction experiment, the adjusted p-values are higher when checking for differential abundances, i.e., we cannot reject the sharp null hypothesis of no differential abundance for the Marvynbriantia genus.”

and elaborated in the Discussion page 23:

“The framework suggested in this paper facilitates a more transparent interpretation of results than standard approaches directly modeling the observed outcome. First, interpretation is only valid within the range of the background covariates of the study population in the respective hypothetical experiment (see their detailed characteristics in Table 1 and Supplementary Figures 2-9). The data do not provide direct information for the ``unmatched'' units. In addition to our pair-matching algorithm, we conducted a sensitivity analysis using a propensity score matching algorithm at the design stage, which led to more matched pairs, and thus a broader range of background covariates values (see Supplementary Table 4). Both matching algorithms do not lead to conflicting results in the smoking prevention experiments. In the air pollution reduction experiment, only the differential abundance analysis does not lead to the same overall conclusion. At this stage, the researcher has to decide to favor a larger number of units or unconfoundedness. We decided to favor unconfoundedness because our research question is nearly untapped. Note that these matching algorithm considerations should be *a priori* specified before any statistical analysis is performed.”

Therefore, I would ask you to carefully address all the reviewers' concerns and especially improve the explanation on how the method actually achieves causal inference. This may require a reorganizing of the manuscript as suggested by Reviewer #2.

As you probably know, applications using the Rubin Causal Model (RCM) have recently been recognized in the field of economics (https://www.nobelprize.org/prizes/economic-sciences/2021/summary/). Here, applying the RCM to rigorously and transparently understand the causal effects of two environmental exposures on the gut microbiome is certainly a strength of our paper. The effect estimates are valid only if our assumptions are correct, especially the unconfoundedness of the assignment mechanism given measured covariates. Furthermore, we believe that more studies, with different designs (e.g., randomized, controlled) and with different populations, are needed to infer causality.

Reviewer 1 - Referee report on "A randomization-based causal inference framework for uncovering environmental exposure effects on human gut microbiota"

This paper describes a matching-based causal inference framework for the microbiome data. The proposed method uses the dataset generated from an observational study to create a pseudo-randomized experiment, based on which the estimates and testing results are endowed with causal interpretation. The paper is well written and the central task, causal inference of microbiome data, is important to the field. There are, however, several points if addressed would make the paper stronger.

• Matching algorithm. The proposed matching algorithm is purely based on the list of covariates that are observed in the study. This is viable when the number of covariates is relatively small but will reduce the number of matched pairs significantly otherwise. In the data example of this manuscript, we can already see the effect of matching on covariates: the original 1,967 subjects were reduced to fewer than 600 subjects after matching. It would be interesting to compare the proposed matching algorithm with the more commonly used propensity score matching (Rosenbaum and Rubin, 1983). Propensity score matching (PSM) takes care of the potential problems associated with high dimensionality of the covariate space and can yield more matched pairs and lead to better power. The tuning process is more straightforward since there's only one tuning parameter in PSM (i.e., the maximal gap in propensity score) (Wu et al., 2018; Ren et al., 2021).

• Tuning approach for the matching algorithm. The manuscript only listed the selected δk but didn't explain how they were derived. Referring to the diagnostic plots is not sufficient. The authors need to provide more details on how they arrive at these δk, especially how they define the metric of covariate balance and whether δk is selected by minimizing this metric.

• Propagation of uncertainty. Although the authors acknowledged that exact alpha- and beta-diversities cannot be recovered from the ASV data and made effort to use statistical models to estimate both diversities, whether the uncertainty of these estimates is propagated into the testing procedure is still of question. I noticed that the authors used standard errors of the estimates as a covariate in the hierarchical regression models, but I'm not convinced this is sufficient for uncertainty propagation. It would be useful to consider non-parametric approaches, such as bootstrap, to address this issue. A potential bootstrap approach could be to first generate bootstrapped data and then carry out the permutations.

• Lack of simulation analysis. The authors proposed a causal inference approach for microbiome data and used it to analyze real data without providing any evidence whether the approach can indeed recover the underlying causal relationships. A simulation study is critical to justify the causal interpretation of the proposed estimates and tests. Simulating microbiome data requires more effort so that the common properties, such as compositionality and zero-inflation, can be preserved. The authors can use resampling approaches (McMurdie and Holmes, 2013) or training a generative model using real data (Ma et al., 2021).

We thank the reviewer for making these excellent and helpful points that we addressed to make the paper stronger.

- *Matching algorithm*: We fully acknowledge that results for sensitivity analysis should be included, as also noted by other reviewers. Therefore, taking the suggestion of Reviewer #1 and Reviewer #3, we have performed a sensitivity analysis comparing the results obtained after our pair-matching strategy to the more commonly used propensity score matching. We have added a new section on page 20 addressing this:

"To assess whether the pair-matching strategy chosen for the design stage could influence the conclusions of this study, we conducted a sensitivity analysis (see Supplementary Sensitivity Analysis section). For that, we implemented the more commonly-used propensity score matching algorithm (Rosenbaum & Rubin, 1983) and obtained matched samples of: 1) 158 participants living in low $PM_{2.5}$ areas and 158 participants living in higher $PM_{2.5}$ areas, and 2) 290 smokers and 290 never smokers (see Supplementary Table 4 and Figures 20-25 for the balance diagnostics). For both hypothetical randomized experiments, using propensity score matching at the design stage results in analyzing more matched samples. The microbial diversity analyses lead to the same conclusion for both experiments despite different design stages. Overall, we also observe small approximate Fisherian p-values after performing the propensity score matching (in the same way we observe small approximate Fisherian p-values with our pair-matching strategy). The test statistics have the same direction and magnitude. For the air pollution reduction experiment, the adjusted p-values are higher when checking for differential abundances, i.e., we cannot reject the sharp null hypothesis of no differential abundance for the Marvynbriantia genus."

and elaborated in the Discussion page 23:

"The framework suggested in this paper facilitates a more transparent interpretation of results than standard approaches directly modeling the observed outcome. First, interpretation is only valid within the range of the background covariates of the study population in the respective hypothetical experiment (see their detailed characteristics in Table 1 and Supplementary Figures 2-9). The data do not provide direct information for the ``unmatched'' units. In addition to our pair-matching algorithm, we conducted a sensitivity analysis using a propensity score matching algorithm at the design stage, which led to more matched pairs, and thus a broader range of background covariates values (see Supplementary Table 4). Both matching algorithms do not lead to conflicting results in the smoking prevention experiments. In the air pollution reduction experiment, only the differential abundance analysis does not lead to the same overall conclusion. At this stage, the researcher has to decide to favor a larger number of units or unconfoundedness. We decided to favor unconfoundedness because our research question is nearly untapped. Note that these matching algorithm considerations should be *a priori* specified before any statistical analysis is performed."

- *Tuning approach for the matching algorithm*: Our threshold values are chosen based on the pre-matching balance of the covariates and subject-matter knowledge. The goal of the design stage is to have a balanced dataset to assume unconfoundedness given the observed covariates. It is a plus to privilege the largest dataset possible with balance. For example, because the "alcohol consumption" variable did not present a large imbalance before matching we decided not to be too stringent on the threshold. The covariates chosen for matching were a priori specified by a co-author not directly involved in the data analysis and based on epidemiological subject-matter knowledge. We clarified in the following statement on page 7:

“At this stage, the objective is to create balanced data subsets for which the plausibility of the "unconfoundedness" assumption is based on a diagnostic of our choice. We choose the thresholds, $\delta_1,...,\delta_7$, according to the pre-matching diagnostic plots of the covariate distributions (see Supplementary Figures 2-7). We privilege a large dataset with balance, while assuring that the created pairs, or in other words "twins", are scientifically plausible, e.g., no male and female could be matched. We assume a covariate to be balanced when its distribution is approximately the same under the exposure vs. not.”

- *Propagation of uncertainty*: We agree with the reviewer that finding powerful test statistics is of great importance in the microbiome data analysis field. However, any test statistic that is a function of the data is valid to test sharp null hypotheses, some are more powerful than others. In this study, we decided to use state-of-the-art test statistics, because we mostly focus on introducing the four-stage framework to the field. We made this clearer in the Introduction with the following sentence on page 3:

“The reason for using this four-stage approach is for the transparency of its assumptions when interpreting results.”

If this is still unclear, we are happy to clarify in another way.

- *Lack of simulation analysis*: The reviewer is correct that simulations can help answer the question underlying causal relationships. We decided to limit the scope of this paper to the implementation of a randomization-based causal inference framework. We test a sharp null hypothesis using test statistics that we believe are powerful from deviations from the sharp null hypothesis. This Fisherian approach differs from the Neymanian approach that estimates a causal estimand and a measure of uncertainty around this estimate. We believe that, given the lack of causal inference literature for our research questions, the first step is to test whether a sharp null hypothesis can be rejected. As a matter of fact, our research group is working on extending the work of this paper and estimating causal effects, which will be supported by simulations as suggested by the reviewer. We introduce this in the Introduction on page 3:

“We do not attempt to provide an estimate of (and uncertainty around) an estimand to avoid relying on assumptions such as the additivity of the treatment effects, asymptotic arguments, or an imputation model, which may be the case when drawing Neymanian (i.e., distribution-based) or Bayesian inferences. This Fisherian approach is a non-asymptotic first step to start shedding light on merely-touched research questions dependent on complex data structures, such as human gut microbiome data.”

We elaborate on this decision in the Discussion and added the references suggested by the reviewer on page 20:

“Throughout the extensive statistical analyses presented in this paper, we have tested sharp null hypotheses of no effect of an intervention on a wide range of gut microbiome outcomes, ranging from high-level microbial diversity estimates to differential genus-genus associations. To do so, we have performed randomization-based inference based on 10,000 permutations. This mode

of inference has been motivated by two reasons: (i) it is difficult to postulate a joint model for the potential outcomes, and thereby provide an estimate of (and uncertainty around) a causal estimand, and (ii) it has been shown that using the actual randomization procedure that led to the observed data helps to report valid Fisher-exact p-values as opposed to p-values relying on approximating null randomization distributions (Bind & Rubin, 2020). As an example, in our mean difference analyses, we found some differences between the null randomization distribution of the test statistic when approximated by permuting the intervention assignment vector and when drawn from the approximating asymptotic distribution (see Supplementary Figures 10 and 11). A natural extension of this study would be to use a Neymanian or Bayesian mode of inference to tackle the same research questions, in such case, simulations should support evidence whether the approach can indeed recover the then estimated causal effects. Simulating microbiome data requires effort so that the common properties, such as compositionality and zero-inflation, can be preserved, but re-sampling approaches (McMurdie & Holmes, 2013) and generative models (Ma et al., 2021) have been developed to achieve this end.

If this is still unclear, we are happy to clarify in another way.

Minor comments.

1. In captions of Fig. 2 and Tables 2-3, the authors suggested one-sided tests were used. However, in the description of the testing procedure in pg. 18, the p-values seem to be calculated with a two-sided alternative
   We thank the reviewer for reading the paper in such detail. Indeed, the description of the p-value calculation was originally misleading. We corrected the descriptions on (now) page 8, as follows:

"In randomization-based inference the goal is to construct the null randomization distribution of a test statistic assuming H_0, T, by computing the values of the test statistic for all possible intervention assignments. Because the number of assignments is very large, we calculate an approximating p-value using N_{iter} iterations, i.e., the proportion of computed test statistics that are as large or larger than the observed test statistic: \frac{1}{N_{iter}} \sum_{l = 1}^{N_{iter}} \mathbb{1}_{T_{l} \geq T^{obs}}, where \mathbb{1}_{T_{l} \geq T^{obs}} = 1 when T_l \geq T^{obs}, and 0 otherwise (for two-sided tests, we obtain the p-values by taking absolute value of T_{l} and T^{obs}, i.e., |T_{l}| and |T^{obs}|)."

2. What might be the reasons for the changes in statistical significance when the data are aggregated into different phylogenic levels for the tests of compositional mean differences? I would imagine that if lower levels (e.g. genus) have strong significance, so does the high levels (e.g. family). This is not the case in the contrast between high vs. low PM exposures.
   We thank the reviewer for pointing out that our p-value reporting lacked a logical explanation. Our results should not be overinterpreted, we see them as exploratory and a first step in an untapped field, in which results reproduction and similar questions analyses are interesting. As Wasserstein et al. (2019) say: "Significance tests and dichotomized *p*-values have turned many researchers into scientific snowbirds, trying to avoid dealing with uncertainty by escaping to a "happy place" where results are either

statistically significant or not. In the real world, data provides a noisy signal. Variation, one of the causes of uncertainty, is everywhere. Exact replication is difficult to achieve." (p.3). Following this logic we tried to establish a clearer results reporting rule in the Results section on page 13:

"Following the American Statistical Association statement (Wasserstein et al., 2016; 2019), we avoid searching for ``statistical significant'' results with a dichotomous approach. To give structure to our results reporting, we reject the sharp null hypotheses of no effect of an environmental intervention when the p-value is lower or equal to 0.1 or, when computed, when the adjusted p-value is lower or equal to 0.2. We are more tolerant with adjusted p-values because multiple comparison adjustments are conservative and our study is exploring a nearly untapped field. Nonetheless, we highly recommend to the readers interested in our research questions or result replication to examine all reported p-values in Figures and Tables, because higher p-values do not mean that an effect is improbable, absent, false, or unimportant (Wasserstein et al., 2019)."

If this is still unclear, we are happy to clarify in another way.

3. A similar question is also for the results of beta-diversity. Why Aitchinson and Jaccard have much larger p-values than the other two beta-diversity for the contrast between high vs. low PM exposures?
   The technical challenges of distance-based approaches, such as beta-diversity, are the reasons why we chose the MiRKAT as a testing approach. We prefered to test four distances instead of choosing one and potentially a non-powerful one. A reason why Aitchison and Jaccard have larger p-values might be due to the fact that these distances have lesser power than UniFrac and Gower in the air pollution reduction experiment setting. We clarify this multiple test statistic choice on page 10:

"Despite the popularity of distance-based approaches, the field of microbiome studies suffers from technical challenges, especially in selecting the best distance. Therefore, we use the suggested microbiome regression-based kernel association test (MiRKAT) (Zhao et al., 2015) that uses a kernel regression and a standard variance-component score test statistic (Lin, 1997). To consider different distance measures, the optimal MiRKAT: tests \mathbf{H_{0,\beta}} for each individual kernel, obtains the p-value for each of the tests, and then adjust for multiple comparison with a p-value with an omnibus test. Instead, we will use a fully randomization-based multiple comparison adjustment method detailed subsequently."

We are open for suggestions from the reviewer to make this analysis method clearer.

4. The authors suggested that the permutations have to preserve the pairing structure (e.g. pg 21, line -4). How is this achieved? I guess it's by permuting the case/control status within each pair?
   We thank the reviewer for pointing out that this statement is unclear. We clarified the sentence on (now) page 12 as follows:

“To generate sampling distributions of the test statistics under H_{0,N}, the intervention and control labels are reassigned 10,000 times to the samples while the matched pair structure is maintained, i.e., the assignment to intervention or control is permuted within each pair.”

5. The multiple testing correction procedure is not well described. For example, the role of raw p-values of the observed statistics is not clear and how do you get the unadjusted p-values for each $T\beta^{h,iter}$?
   We are sorry that the multiple testing correction procedure was not well described. We gave more details to the procedure on page 11:

“

1. Calculate for each hypothesis h, an unadjusted p-value for the observed test statistic by taking the proportion of computed test statistics that are as large or larger than the observed test statistic. This procedure is detailed in the introduction of the Statistical analysis stage section. Also, for each hypothesis h, h = 1,..,H, record the T_{\beta}^{h, iter} = (T_{\beta}^{1, 1}, ..., T_{\beta}^{H, N_{iter}}), where iter = 1,...,N_{iter}.
2. For each h and each iteration iter, calculate an unadjusted randomization-based p-value, with T_{\beta}^{h, iter} as the observed test statistic. For each iter, record the minimum p-value of the H p-values.
3. The repetitions of Step 2 capture the joint randomization distribution of the test statistics and thus, of the unadjusted p-values.
4. To calculate the adjusted p-values for the observed test statistics, for each h, take the proportion of ``minimum p-values'' (recorded in Step 2) that are less than or equal to its unadjusted p-value calculated in Step 1.

”

If this is still unclear, we are happy to clarify in another way.

6. How do you handle zeroes in C_i^a when calculating L^a_i ?
   When calculating L^a_i , we handle zeros by using the method of Cao et al. (2018) who introduced this test statistic, i.e., before the centered log-ratio transformation, pseudo counts of 0.5 are added to all zero entries.

7. Typos.
   – pg. 16, line -5, “guaranty” should be “gaurantee”.
   Corrected.
   – pg. 19, line -15, “H0,B” should be “H0,R”.
   Corrected.
   – pg. 20. “In our case a hypothetical ...” is not a complete sentence.
   Corrected.
   – pg. 20. In the definition of g(Ci), 1/a should be 1/A.
   Corrected.

Reviewer 2 - Review of: “A randomization-based causal inference framework for uncovering environmental exposure effects on human gut microbiota” by Sommer et al.

The paper focuses on the analysis of a microbiome data set from the KORA cohort study. The goal was to consider the question of whether or not (a) air pollution reduction and (b) smoking cessation changed the microbiome. While the standard microbiome analysis is performed on the data sets, the strength of the paper lies in applying the Rubin Causal Model to the data sets to go beyond correlational and associational explorations. This is done using the carefully laid out 4-step process consisting of conceptual, design, analysis and summary. The statistical work is done well, except for the language about p-values and accepting or rejecting the null hypotheses.

The organization of the paper is awkward. The causal analysis is confined to the discussion section. All of this should belong to the Methods section, as done here. But the results are not discussed in the Results section, and no discussion is provided.

We apologize that the original organization of the paper was awkward. This might be due to the interdisciplinary (epidemiology, microbiome research, and statistics) nature of this paper and the different organization rules respective to research fields. We have reorganized the sections as follows:

1. Introduction: we first give an overview of the literature linked to our research questions and then introduce the reasons for our statistical methods choices.

2. Methods: we explain in detail the 4-steps approach used to tackle our research questions.

3. Results: we present how we implemented the methods explained in the previous section for our two hypothetical randomized experiments.

4. Discussion: we first discuss the results presented in the above section, then elaborate on the statistical framework we used for our analyses and suggest statistical and epidemiological extensions of our work.

We are open to further suggestions on how the paper structure could be less awkward.

Overall, this is a good paper with solid analyses and deserves to be published.

Major Comments

1. I understand the instinct to follow the ASA suggestion to avoid the arbitrary p-value threshold of 0.05. However, it does not eliminate the arbitrariness from their decisions to decide when a p-value is “small” or “low”, and therefore, significant. On p4, the authors claim to “only describe the Fisherian p-values”, but the paper does accept or reject several null hypotheses with no uniformity or logic to dictate those action(s). For example, Fig. 2 suggests rejecting the null hypotheses of no difference in richness and diversity due to air pollution, but the values for smoking are hardly small (about 0.15), and yet they are being rejected. When is it “small” and who decides and on what basis? Additionally, I suspect that there are thresholds built into tools like SPIEC-EASI, among

others that they have employed. A statement on p13 states that they only reject null hypotheses and that it indicates further study and not that they are different. I find this logic strange. With no explicit threshold, the explanations seem murky and arbitrary. Arbitrarily calling for “further scrutiny” is very unsatisfying. Can the authors provide an “algorithm” for when they reject and when they call for further study?

We thank the reviewer for pointing out that our p-value reporting lacked a logical explanation. This might be due to the fact that we do not want to overinterpret our results that we see as exploratory and a first step in an untapped field, in which results replication of similar questions would be interesting. As Wasserstein et al. (2019) say: “Significance tests and dichotomized *p*-values have turned many researchers into scientific snowbirds, trying to avoid dealing with uncertainty by escaping to a “happy place” where results are either statistically significant or not. In the real world, data provides a noisy signal. Variation, one of the causes of uncertainty, is everywhere. Exact replication is difficult to achieve.” (p.3). Following this logic we tried to establish a clearer results reporting rule in the Results section on page 13:

“Following the American Statistical Association statement (Wasserstein et al., 2016; 2019), we avoid searching for ``statistical significant'' results with a dichotomous approach. To give structure to our results reporting, we reject the sharp null hypotheses of no effect of an environmental intervention when the p-value is lower or equal to 0.1 or, when computed, when the adjusted p-value is lower or equal to 0.2. We are more tolerant with adjusted p-values because multiple comparison adjustments are conservative and our study is exploring a nearly untapped field. Nonetheless, we highly recommend to the readers interested in our research questions or result replication to examine all reported p-values in Figures and Tables, because higher p-values do not mean that an effect is improbable, absent, false, or unimportant (Wasserstein et al., 2019).”

If this is still unclear, we are happy to clarify in another way.

2. Shouldn’t there be a discussion on the anomaly of alpha and beta diversity giving two (marginally) different messages?
   We thank the reviewer for carefully comparing the results of the alpha and beta diversity analyses. With the newly detailed logic, the null hypotheses can be rejected in both analyses. But we are open to further suggestions on how we could better present these results.

3. The “Differential genus-genus associations” were a bust. The p-values were not really small, but two of the edges were arbitrarily called out as significant.
   Based on the logic newly detailed and inserted in the text, we believe that the differential genus-genus analysis can remain. We do not mention any results to be “significant” in this paper because we believe it can be a misleading statement. We are open for a suggestion if the reviewer does not share our opinion.

4. I have suggested many of these statements about rejecting the null hypotheses to be tempered. These suggestions should also appropriately modify the discussion as well, where these statements have been repeated.

Providing tempered statements on the interpretation of p-values is a task that every statistician should take seriously. Thanks to the reviewer, we attempted to make our reporting logic clearer. If this is still unclear, we are happy to clarify in another way.

Minor Comments

We thank the reviewer for taking the time for giving us these “minor” but worthwhile correcting comments.

p1, Abstract In sentence 2, move the word “however” to the start of the sentence.
Moved.

p6, line 3 I suggest the statement be modified to say something to the effect that “The Fisherian p-values for rejecting the null hypotheses for the smoking prevention data is not strong, and other measures will be explored.”
Based on the logic newly detailed and inserted in text, we believe that the original statement can remain. We are open for a suggestion if the reviewer does not share our opinion.

p6, Between-subject variation Same problem as above. This time the roles are switched. And I suggest taking a similar strategy and making a similar statement as above, but about beta diversity with air pollution.
Based on the logic newly detailed and inserted in text, we believe that the original statement can remain. We are open for a discussion if the reviewer does not share our opinion.

p6, Between-subject variation Delete the phrase “p-values are small” or else provide a clear logic for why.
We deleted the words “p-values are small”.

p7 Rewrite the phrase “p-values are low” as “p-values are lower at the ASV level than at the higher levels of the taxonomy”.
We rewrote the phrase as suggested.

p7, 7th line from bottom Delete “do not”. I believe you want to reject here for the 11 genera.
Corrected.

p8, 1st line Replace “high-level” by “deeper” or “greater”.
We removed the word “high-level”.

p10 I want to see a clear statement that states that the evidence for rejecting the null hypothesis was very marginal, but that along the edges the ones for which the greatest support was available were the ones they mention.
Based on the logic newly detailed and inserted in text, we believe that the original statement can remain. We are open for a suggestion if the reviewer does not share our opinion.

p11, Discussion Delete the phrase “Due to the interdisciplinary (epidemiology, microbiome research, and statistics) nature of this paper”.
Deleted.

p12, line 2 Rejecting the sharp null hypothesis for the smoking study has inadequate support.
Based on the logic newly detailed and inserted in text, we believe that the original statement can remain. We are open for a suggestion if the reviewer does not share our opinion.

p12, line 19 replace “depict” by “observe”
Replaced.

p12, line 20 replace “genera than reported by Lee” by “genera that were reported by Lee” p14, line 4 replace “direction than” by “direction as”.
Replaced.

p14, line 4 from bottom replace “libraries are then” by “libraries were then”
Replaced.

p15, line 14 replace “16s” by “16S”
Replaced.

p22, line 8 replace “cautiousness” by “caution”
Replaced.

Reviewer #3: The paper introduces a causal inference framework to investigate the treatment effect of environmental factors on the human microbiome. The core idea is to use matched pairs to balance the pre-exposure characteristics of participants and then to use randomization-based inference. The authors illustrated their framework on the German KORA cohort study, specifically, the effects of air pollution reduction and smoking prevention on the human gut microbiome. The paper is easy to follow and includes various analyses in the proposed framework. However, the framework is nothing but just matching, where sensitivity analysis is an essential part. The authors mentioned sensitivity analysis in the Discussion, but the reviewer doesn't think that is enough. Results for sensitivity analysis should be included, as well-known factors that are associated with the microbiome, such as diet and medication, were not used in matching. It is also not clear how their results differ from the analyses with covariate adjustments, which is commonly performed in microbiome studies. The authors mentioned the unreliability of regression with covariate adjustment in the Discussion, but matching is also not reliable if there are unmeasured covariates that have confounding effects. It would be very instructive if the authors could demonstrate that matching is more reliable than covariate adjustment in the microbiome study.

- We thank the reviewer for their comments and fully acknowledge that results for sensitivity analysis should be included, as also noted by other reviewers. Therefore, taking the suggestion of Reviewer #1 and Reviewer #3, we have performed a sensitivity analysis comparing the results obtained after our pair-matching strategy to the more commonly-used propensity score matching. We have added a new section on page 20 addressing this:

"To assess whether the pair-matching strategy chosen for the design stage could influence the conclusions of this study, we conducted a sensitivity analysis (see Supplementary Sensitivity Analysis section). For that, we implemented the more commonly-used propensity score matching algorithm (Rosenbaum & Rubin, 1983) and obtained matched samples of: 1) 158 participants living in low $PM_{2.5}$ areas and 158 participants living in higher $PM_{2.5}$ areas, and 2) 290 smokers and 290 never smokers (see Supplementary Table 4 and Figures 20-25 for the balance diagnostics). For both hypothetical randomized experiments, using propensity score matching at the design stage results in analyzing more matched samples. The microbial diversity analyses lead to the same conclusion for both experiments despite different design stages. Overall, we also observe small approximate Fisherian p-values after performing the propensity score matching (in the same way we observe small approximate Fisherian p-values with our pair-matching strategy). The test statistics have the same direction and magnitude. For the air pollution reduction experiment, the adjusted p-values are higher when checking for differential abundances, i.e., we cannot reject the sharp null hypothesis of no differential abundance for the Marvynbriantia genus."

and elaborated in the Discussion page 23:

"The framework suggested in this paper facilitates a more transparent interpretation of results than standard approaches directly modeling the observed outcome. First, interpretation is only valid within the range of the background covariates of the study population in the respective hypothetical experiment (see their detailed characteristics in Table 1 and Supplementary Figures 2-9). The data do not provide direct information for the ``unmatched'' units. In addition to our pair-matching algorithm, we conducted a sensitivity analysis using a propensity score

matching algorithm at the design stage, which led to more matched pairs, and thus a broader range of background covariates values (see Supplementary Table 4). Both matching algorithms do not lead to conflicting results in the smoking prevention experiments. In the air pollution reduction experiment, only the differential abundance analysis does not lead to the same overall conclusion. At this stage, the researcher has to decide to favor a larger number of units or unconfoundedness. We decided to favor unconfoundedness because our research question is nearly untapped. Note that these matching algorithm considerations should be *a priori* specified before any statistical analysis is performed."

- We completely agree with the reviewer that diet and medication are well-known factors that are associated with the microbiome. We clarified in the following paragraph on page 8:

"It is well known that diet has an influence on the gut microbiome and future studies on the gut should include dietary intake data in their analysis (Singh et al, 2017, Johnson et al., 2020). In our study, we only have access to dietary intake data for a portion of our samples, therefore we examine balance diagnostics in usual nutrient intake after matching in order to maintain a large data set before matching. Supplementary Figures 8 and 9 show that after matching, our intervention and control units (in both hypothetical experiments) do not exhibit imbalance with respect to the following food items: potatoes/roots, vegetables, legumes, fruits/nuts, dairy products, cereal products, meat, fish, egg products, fat, and sugar. In the same way, we checked for covariate balance after matching for medication intake, also a well-known confounder in human gut microbiome studies. Supplementary Figures 4 and 7 show that after matching, our intervention and control units (in both hypothetical experiments) do not exhibit imbalance with respect to medication intake."

- We agree with the reviewer that, when confounders are unmeasured, a design stage with a matching strategy cannot undergo unmeasured confoundedness issues. This is why an assumption of the Rubin Causal Model is that the assignment mechanism is unconfounded given measured covariates. We made this assumption clearer on page 7:

"The aim of our pair-matching strategy is to achieve balance in background covariates distributions as it is expected, on average, in randomized experiments. This strategy attempts to create exchangeable groups as if the exposure was randomly assigned to each participant, to guarantee exposure assignment is not, on average, confounded by the measured background covariates. The exposure assignment mechanism determines which units receive which exposure; in other words, which potential outcomes are observed and which are missing (Imbens & Rubin, 2015). Unconfoundedness of the assignment mechanism is a key assumption of the Rubin Causal Model."

- The unreliability of regressions when a design stage is omitted in observational studies has already extensively been shown and discussed in various fields (Cochran & Rubin, 1973; Heckman, 1998; Rubin 2001), including the field of study of this paper environmental epidemiology (Bind and Rubin, 2017) and the field of one of the latest Nobel Prize Winner: Labor Economics (Imbens & Rubin, 2015). Demonstrating that matching is more reliable than covariate adjustment in our microbiome study is beyond the scope of this paper. In this paper, we are introducing the Rubin Causal Model and randomization-based inference to the microbiome data analysis field, because its

transparent assumptions can be beneficial to avoid extrapolation when interpreting results. We made this clearer in the Introduction with the following sentence on page 3:

“The reason for using this four-stage approach is for the transparency of its assumptions when interpreting results.”

If this is still unclear, we are happy to clarify in another way.

Minor:

Column titles in Table 1 are switched.

We very much appreciate that the reviewer went through the paper in such detail. Would it be possible to give us more detail on the column title that is being switched in order to locate the mistake?

It seems rather subjective in determining thresholds for covariates in matching. How was the threshold for each covariate determined?

Their chosen values are chosen based on the pre-matching balance in covariates. The goal of the design stage is to have a balanced dataset to assume unconfoundedness. It is a plus to privilege the largest data set possible with balance. For example, because the “alcohol consumption” variable did not present a large imbalance before matching we decided not to be too stringent on the threshold. We clarified in the following statement on page 7:

“At this stage, the objective is to create balanced data subsets for which the plausibility of the "unconfoundedness" assumption is based on a diagnostic of our choice. We choose the thresholds, $\delta_1,...,\delta_7$, according to the pre-matching diagnostic plots of the covariate distributions (see Supplementary Figures 2-7). We privilege a large dataset with balance, while assuring that the created pairs, or in other words "twins", are scientifically plausible, e.g., no male and female could be matched.”
