# Supplementary material for: A randomization-based causal inference framework for uncovering environmental exposure effects on human gut microbiota
Source: PLoS Comput Biol. 2022 May 9;18(5):e1010044. doi: 10.1371/journal.pcbi.1010044 (PMC9129050; doi:10.1371/journal.pcbi.1010044)
Supplement: Attachment — Submitted filename: review2.pdf [file pcbi.1010044.s005.pdf]

Review of: "A randomization-based causal inference framework for uncovering environmental exposure effects on human gut microbiota"

by Sommer et al.

I reiterate that the strength of the paper lies in carefully applying the **Rubin Causal Model** to the data sets to go beyond correlational and associational explorations. In order to weigh in on the suggestion that the work might be nothing more than plain "matching", let me say that one way to think about Rubin Causal Model is that it allows us to "simulate" a randomized clinical trial on the data set, which is achieved by the matching step. The more critical thing to note is that because it is a virtual simulation, the process allows us to *randomly* pick a large number of matchings from the space of all good matchings, thus simulating multiple trials. In this paper, 10,000 simulations were performed. I believe it is the right way to apply the well-known Rubin Causal Model and is a reasonable approach for this data set. As pointed out by the authors, this model has been widely accepted and adopted (and also recognized by the recent Nobel Prize for Imbens) in econometrics and other domains.

This work enhances the value of existing large-cohort observational data sets that may have been collected for a different purpose. Overall, this is a good paper with meticulous analyses and deserves to be published.

Major Comments

1. The suggested model explains that air pollution introduces particulate matter which enters the lungs and then potentially gets into the gut because of mucocilliary clearance and therefore comes in contact with the gut microbiome. However, the model for how smoking might come in contact with the gut microbiome is not well laid out. Could the authors add some clarity to this point? Also, even with particulate matter, couldn't there be a simple explanation with the fact that breathing through the mouth provides an entry point for particulate matter and smoke into the alimentary canal and consequently into the gut?

2. In the *Bioinformatics* section of "Methods", the authors list the steps in the DADA2 pipeline. But the reader may be unfamiliar with how phylogenetic analysis fits into an ASV-based analysis. The authors do not clearly state the connection.

3. I have assumed that the cohorts for the air pollution and smoking were distinct. If there was any overlap, it would be useful to know the size of the overlap. A comment on this matter would be useful to the reader.

4. The authors should use the term "bacterial taxa" instead of "bacteria", where appropriate. Please make a global change. Not every occurrence has been tracked in the comments below.

5. The careful wording for the p-value discussion is appreciated.

6. In Supplementary figures 2-7, it was unclear of the distributions shown are an average of each cohort over all repetitions or if it was computed by pooling all the repetitions. Please clarify.

7. Italicize all taxa names at species, genera and family levels, as is the accpeted convention. Change this globally, including in figures, captions, and supplementary material.

Minor Comments

Abstract, line 14 Italicize all taxa names at species, genera and family levels, as is the accpeted convention.

p5, *Bioinformatics* Add a sentence or two that states what is done with the results of the DADA2 pipeline and why, along with why it needs a whole genome alignment method.

p6, "Conceptual stage" After Eq. (1), replace "bacterium" by "bacterial taxon" in several places. Replace "bacteria-b" by "taxon-b".

p6, line 2 Replace "Rubin" by "Rubin model" since the reference has more than one author.

p6, "Observed outcomes measurement" ASVs are targeting strains, not just species. Suggest replacing "species" by "strains" on line 2.

p7, lines 7 and 11 Double quotes go in the wrong direction.

p10, top of page Replace "bacteria" by "bacteria taxa" or just "taxa" on lines 1, 2, 3, 5.

p10, "Choice of test stat" Replace "by" by "by Willis et al."

p11, line 1 The authors refer to UniFrac as the "proper distance metric" without an explanation or a reference. Please provide. In the next sentence they say "the same applies" and it is unclear what applies. Rephrase to make it more clear.

p11, line 17 Pluralize "Step 1-4".

p11, Step 1 Define notation $T_{\beta}^{h,iter}$.

p12, *Diff Abundance* Replace "bacteria" by "bacteria taxa". Also Section 4.

Sec 2, last line Missing period.

p16 Italicize genus name "*Marvinbryantia*" and all family names that follow. Also on page 17, page 19, 20, ...

Supplementary material Replace "bacteria" by "bacteria taxa" or just "taxa" in Suppl Table 7 and caption.

p20, last line Authors say "we cannot reject the sharp null hypothesis of no differential abundance for the Marvynbriantia genus (see Supplementary Table 7)." But Table 7 does not mention genus *Marvynbriantia.*
