# Supplementary material for: A randomization-based causal inference framework for uncovering environmental exposure effects on human gut microbiota
Source: PLoS Comput Biol. 2022 May 9;18(5):e1010044. doi: 10.1371/journal.pcbi.1010044 (PMC9129050; doi:10.1371/journal.pcbi.1010044)
Supplement: Attachment — Submitted filename: Reviewer_comments_Sommer_roundTWO.pdf [file pcbi.1010044.s006.pdf]

We thank the Guest Editor and three reviewers for their second revision round and their helpful comments. We believe our paper has been greatly improved by incorporating the additional thoughtful feedback provided. More details on changes can be found below, with our responses recorded in blue.

Guest Editor's comments:

I would like to thank the authors for their revised submission and the reviewers for their detailed and insightful comments on it. I'm enjoying being guest editor for this paper as it prompted me to read up on Rubin causality, and I have learned a lot from working through paper and comments.

Reviewer #2 had much praise for the improved paper, and I also agree with them that the author's reorganization of the paper has improved readability greatly. Reviewers #1 and #3 still have a few major concerns. Overall, I tend to agree with reviewer #2 that the paper is now in a good shape, pending the authors addressing the numerous minor comments made by all three reviewers. I would ask you to go through them carefully and amend and clarify the text accordingly.

The authors have rightfully pointed out that by applying Rubin's causal model to high-dimensional data and complex hypotheses that intermingle the dimensions (such as diversity measures) they are the first to bridge two subjects so far not connected, and that this paper is therefore a first step and cannot be expected to address every aspect of the topic. In this light, I would say that some of the Reviewers' remaining major concerns merit a paper in its own right and would not need to be addressed here, provided the necessity for such further research is mentioned in the discussion.

> We thank the Guest Editor for its kind assessment of our work.
>
> We agree, this paper is a first step in bridging two topics so far not connected. As a matter of fact, two members of our research group are working on extending the work of this paper and will cautiously take the pertinent comments of the reviewers into account. Therefore, in this second revision round, we follow the advice of the guest editor and amend our paper according to all reviewers' minor comments, as well as most of the major comments, while avoiding to increase the length and deviating from the focus of our paper.

Specifically:

- Reviewer #1 is certainly right that PSM is the only workable solution if one has many covariates, but the present experiment has only few -- so we can leave this issue to whoever is the first to actually face a situation with many covariates. For the present case of few covariates, the authors consider it obvious that individual matching beats PSM, the Reviewer doubts that. Hence, this statement should be either elaborated or dropped.

Thank you very much for letting us know that we came across as favoring pair-matching vs. PSM. The matching algorithm used at the design stage is at the discretion of the researcher and we did not want to say that one algorithm is better than the other, but we wanted to show that the algorithm choice, in our case, does not tremendously affect our results interpretations. We tried to make this clearer in the Discussion p. 18:

> At this stage, the researcher can decide between a larger number of units or more similar groups of units to compare. When designing our hypothetical experiment, we chose a pair-matching strategy, because it creates similar pairs of participants based on subject-matter knowledge. For example, the number of females and males in the intervention and control groups is identical after pair-matching, whereas with propensity score matching, these numbers slightly differ (see Table 4 and

> Supplementary Table 4). Note that the matching algorithm considerations should be *a priori* specified before any statistical analysis is performed. Nevertheless, we were satisfied to observe that our conclusions did not dramatically change.

- I do not quite agree with Reviewer #1 on the need for simulations to ensure that null distributions are as expected. A simulation cannot help us to assess whether matching properly remedies confounding. However, once we assume there to be no confounding, we are in the same situation as in an actual randomized study. The question whether the tests employed by the authors are appropriate for randomized studies or controlled experiments in metabolomics have been discussed thoroughly in the papers that introduced these tests to the field of metabolomics -- and hopefully checked by simulations there. So, citing existing literature seems sufficient to me here.

We agree that simulation studies are important, but they will be performed by two members of our teams in a subsequent paper.

- Reviewer #3 criticizes the lack of a quantitative analysis of sensitivity to confounding. I imagine that here the bigger issue is not residual confounding to the (very few) known covariates but the influence of unknown/unrecorded covariates. Hence, while ideally, the authors would add a quantitative sensitivity analysis in the Reviewer's sense, a qualitative discussion of the risk of (a) insufficient compensation for the known confounders and (b) unaccounted confounders should be sufficient, too.

We agree that analyzing the sensitivity of p-values could be interesting to assess the plausibility of the unconfoundedness assumption. But, this would merit a paper in itself and requires subject-matter knowledge on the probability of the binary exposure (i.e., smoking or air pollution) given the observed and unobserved background covariates on the binary exposure (i.e., smoking or air pollution). We make this clearer on p. 17-18 of the Discussion:

> Even though we made sure that the observed potential confounding covariates are fairly balanced, there could still be imbalances in other unobserved background covariates, which could have an effect on our results. In such cases, Rosenbaum (2010) has recommended to consider sensitivity analyses of how the Fisher-exact p-value would change, had the intervention assignment been plausibly different, see also Bind & Rubin (2020). Subject-matter knowledge on the probability of the binary exposure (i.e., smoking or air pollution) given the observed and unobserved background covariates should guide the plausible range of ``sensitivity'' p-values and the reason why they could deviate from the p-value calculated based on the assumed hypothetical intervention assignment. This idea provides material for an extension of the framework presented in this study.

- Regarding Reviewer #2's second comment, on comparing matching on a subset with accounting and using all samples: This is a question of gaining optimal power, not of ensuring correctness of inference. As the present paper does not claim to be the final word on the topic, such ways of optimizing inferential power are arguably beyond its scope, so we can leave this discussion to some future work.

45 units are in both cohorts. We included a comment on this fact in the *Characteristics of study population* section, but we agree that this discussion can be left to future work and thank the Guest Editor for giving us his honest opinion.

---8<--

Reviewer's Responses to Questions

Comments to the Authors:

Please note here if the review is uploaded as an attachment.

Reviewer #1: The authors have done a good job of responding to the previous comments and suggestions. However, two responses need further clarifications.

1. Comparisons to propensity score matching (PSM). The sensitivity analysis suggests that PSM seems to generate results consistent with those from matching on covariates while tends to get more matching pairs and thus lead to smaller approximate p-values. This comparison indicates PSM might be a better approach for matching. However, in the revised Discussion section, the authors claim that the proposed approach is favored over PSM since "unconfoundedness" should be prioritized. I'm not completely clear why matching on covariates directly would achieve better unconfoundedness compared to PSM, especially considering the fact that a propensity score model can incorporate high dimensional potential confounders which to me appears as a more flexible tool to adjust for confounding. The authors should clarify more on this point.

Thank you very much for letting us know that we came across as favoring pair-matching vs. PSM. The matching algorithm used at the design stage is ideally performed by a statistician not involved in the subsequent statistical analysis stage, in order to avoid model cherry-picking. Here, we did not want to say that one algorithm is better than the other, but we wanted to show that the algorithm choice, in our case, does not tremendously affect our results interpretations. We tried to make this clearer in the Discussion p. 18:

> At this stage, the researcher can decide between a larger number of units or more similar groups of units to compare. When designing our hypothetical experiment, we chose a pair-matching algorithm, because it creates similar pairs of participants based on subject-matter knowledge. For example, the number of females and males in the intervention and control groups is identical after pair-matching, whereas with propensity score matching, these numbers slightly differ (see Table 4 and Supplementary Table 4). Note that the matching algorithm considerations should be *a priori* specified before any statistical analysis is performed. Ideally, the design stage should be conducted by a statistician who is not involved in the subsequent statistical analysis stage.

2. I still believe some form of simulations should be done in order to validate the proposed approach. As the authors pointed out, understanding the causal effects of microbiome is nearly untapped and we cannot easily transfer our knowledge of the statistical properties of matching algorithms in regular univariate outcome scenarios to microbiome data. I understand that the goal of the approach is to provide exploratory analysis and hard thresholding of p-values for decision making is somewhat questionable. But we at least need to know whether the p-values from the approach under the null has expected behaviors (e.g., uniform distribution) and whether the approach after multiple correction has sufficient power to detect causal effects when the data generation process is known (e.g. in a simulation study). Even a simple low-dimensional example with several homogeneous treatment effect would make the paper much stronger.

We agree that conducting a sensitivity analysis for the p-values is interesting, but this would merit a paper in itself and require subject-matter knowledge on the probability of the binary exposure (i.e., smoking or air pollution) given the observed and unobserved background covariates. We make this clearer on p. 17-18 of the Discussion:

> Even though we made sure that the observed potential confounding covariates are fairly balanced, there could still be imbalances in other unobserved background

> covariates, which could have an effect on our results. In such cases, Rosenbaum (2010) has recommended to consider sensitivity analyses of how the Fisher-exact p-value would change, had the intervention assignment been plausibly different, see also Bind & Rubin (2020). Subject-matter knowledge on the probability of the binary exposure (i.e., smoking or air pollution) given the observed and unobserved background covariates should guide the plausible range of ``sensitivity'' p-values and the reason why they could deviate from the p-value calculated based on the assumed hypothetical intervention assignment. This idea provides material for an extension of the framework presented in this study.

The multiple correction approach having sufficient power to detect causal effects is suggested in the paper introducing the procedure. We made it clearer on p. 6:

> We follow the fully randomization-based procedure for multiple comparisons adjustments suggested by Lee et al. (2017), which is directly motivated by the intervention assignment actually used in the experiment. This procedure has been suggested to have sufficient power to detect causal effects (Lee et al., 2017).

Reviewer #2: I reiterate that the strength of the paper lies in carefully applying the Rubin Causal Model to the data sets to go beyond correlational and associational explorations. In order to weigh in on the suggestion that the work might be nothing more than plain "matching", let me say that one way to think about the Rubin Causal Model is that it allows us to "simulate" a randomized clinical trial on the data set, which is achieved by the matching step. The more critical thing to note is that because it is a virtual simulation, the process allows us to randomly pick a large number of matchings from the space of all good matchings, thus simulating multiple trials. In this paper, 10,000 simulations were performed. I believe it is the right way to apply the well-known Rubin Causal Model and is a reasonable approach for this data set. As pointed out by the authors, this model has been widely accepted and adopted (and also recognized by the recent Nobel Prize for Imbens) in econometrics and other domains.

This work enhances the value of existing large-cohort observational data sets that may have been collected for a different purpose. Overall, this is a good paper with meticulous analyses and deserves to be published.

We thank Reviewer #2 for its kind assessment of our work. Indeed, we believe it is the right way to apply the Rubin Causal Model. In 2018, Rubin published the 4-step procedure described in this paper (Bind & Rubin, (2018)) .

Major Comments

1. The suggested model explains that air pollution introduces particulate matter which enters the lungs and then potentially gets into the gut because of mucociliary clearance and therefore comes in contact with the gut microbiome. However, the model for how smoking might come in contact with the gut microbiome is not well laid out. Could the authors add some clarity to this point? Also, even with particulate matter, couldn't there be a simple explanation with the fact that breathing through the mouth provides an entry point for particulate matter and smoke into the alimentary canal and consequently into the gut?

Thank you for letting us know that the models of PM and smoking exposure of the gut were unclearly explained. We gave more details as follows in the Introduction on p. 2:

> Several potential pathways explain how particles affect human health. The gut is exposed to PM through: (i) mucociliary clearance, i.e., the self-cleaning mechanism of the bronchi, inducing inhaled PM to be cleared from the lungs to the gut, and (ii) oral route exposure, when food and water is contaminated by PM prior to being ingested or in the alimentary canal via inhalation (Moller et al., 2004; Beamish et al., 2011).

and p. 3:

> The chemical mixture of cigarette smoke inhaled into the lungs has an effect on blood markers that, in turn, interact with the gut. Another pathway is that the toxicants of cigarette smoke swallowed into the gastrointestinal tract induce gastrointestinal microbiota dysbiosis via antimicrobial activity and regulation of the intestinal microenvironment (Gui et al., 2021).

2. In the Bioinformatics section of "Methods'', the authors list the steps in the DADA2 pipeline. But the reader may be unfamiliar with how phylogenetic analysis fits into an ASV-based analysis. The authors do not clearly state the connection.

Thank you for pointing out that the Bioinformatics paragraph was lacking information. We added the following sentences:

> The result of the DADA2 pipeline is two datasets: (i) a ASV count dataset, where each row specifies how often an ASV was sequenced and (ii) a taxonomic

> assignment dataset, where each row specifies the taxonomic names of an ASV. It is common to create a phylogenetic tree of the ASVs to later on calculate microbial diversity measures such as the DivNet (Willis et al., 2020) and UniFrac (Lozupone et al., 2005) (see the Statistical analysis stage of Methods Section 2).

3. I have assumed that the cohorts for air pollution and smoking were distinct. If there was any overlap, it would be useful to know the size of the overlap. A comment on this matter would be useful to the reader.

Thank you for pointing out that it was unclear whether the air pollution and smoking matched pair sets had some overlap or not. Indeed, 45 units are in both cohorts. We included a comment on this fact in the *Characteristics of study population* section.

4. The authors should use the term "bacterial taxa'' instead of "bacteria", where appropriate. Please make a global change. Not every occurrence has been tracked in the comments below.

Corrected.

5. The careful wording for the p-value discussion is appreciated.

Thank you.

6. In Supplementary figures 2-7, it was unclear if the distributions shown are an average of each cohort over all repetitions or if it was computed by pooling all the repetitions. Please clarify.

Supplementary Figures 2-7 show the distribution of the background covariates in the original dataset and after matching. The repeated intervention assignments do not yet play a role at this design stage.

7. Italicize all taxa names at species, genera and family levels, as is the accepted convention. Change this globally, including in figures, captions, and supplementary material.

Corrected.

Minor Comments

Abstract, line 14 Italicize all taxa names at species, genera and family levels, as is the accepted convention.

Corrected.

p5, Bioinformatics Add a sentence or two that states what is done with the results of the DADA2 pipeline and why, along with why it needs a whole genome alignment method.

Thank you for pointing out that the Bioinformatics paragraph was lacking information. We added the following sentences:

> The result of the DADA2 pipeline is two datasets: 1) a ASV count dataset, where each row specifies how often an ASV was sequenced, and 2) a taxonomic assignment dataset, where each row specifies the taxonomic names of an ASV. It is common to create a phylogenetic tree of the ASVs to later on calculate microbial diversity measures such as the DivNet (Willis et al., 2020) and UniFrac (Lozupone et al., 2005) (see the Statistical analysis stage of Methods Section 2).

p6, "Conceptual stage" After Eq. (1), replace "bacterium" by "bacterial taxon" in several places. Replace "bacteria-b" by "taxon-b".

Corrected.

p6, line 2 Replace “Rubin” by “Rubin model” since the reference has more than one author.

Corrected.

p6, ”Observed outcomes measurement” ASVs are targeting strains, not just species. Suggest replacing “species” by “strains” on line 2.

Corrected.

p7, lines 7 and 11 Double quotes go in the wrong direction.

Corrected.

p10, top of page Replace “bacteria” by “bacteria taxa” or just “taxa” on lines 1, 2, 3, 5.

Corrected.

p10, ”Choice of test stat” Replace “by” by “by Willis et al.”

Corrected.

p11, line 1 The authors refer to UniFrac as the “proper distance metric” without an explanation or a reference. Please provide. In the next sentence they say “the same applies” and it is unclear what applies. Rephrase to make it more clear.

Corrected.

p11, line 17 Pluralize “Step 1-4”.

Corrected.

p11, Step 1 Define notation T h,iter β .

Corrected.

p12, Diff Abundance Replace “bacteria” by “bacteria taxa”. Also Section 4.

Corrected.

Sec 2, last line Missing period.

Corrected.

p16 Italicize genus name “Marvinbryantia” and all family names that follow. Also on page 17, page 19, 20, ...

Corrected.

Supplementary material Replace “bacteria” by “bacteria taxa” or just “taxa” in Suppl Table 7 and caption.

Corrected.

p20, last line Authors say “we cannot reject the sharp null hypothesis of no differential abundance for the Marvynbriantia genus (see Supplementary Table 7).” But Table 7 does not mention genus Marvynbriantia.

Corrected.

Reviewer #3: The authors made a great effort to address reviewers' comments, but the authors' responses are not quite satisfactory.

1. In causal inference for observational studies, "sensitivity analysis" typically refers to a method that assesses the magnitude of violations from unconfoundedness (See Imbens & Rubin, 2015). The propensity score matching is another way of matching and needs an assessment of potentially unmeasured confounding effects, like every method in causal inference for observational studies. An assessment of unconfoundedness should be included, and it would make this paper more appealing.

We agree that analyzing the sensitivity of p-values could be interesting to assess the plausibility of the unconfoundedness assumption. But, this would merit a paper in itself and requires subject-matter knowledge on the probability of the binary exposure (i.e., smoking or air pollution) given the observed and unobserved background covariates on the binary exposure (i.e., smoking or air pollution). We make this clearer on p. 17-18 of the Discussion:

> Even though we made sure that the observed potential confounding covariates are fairly balanced, there could still be imbalances in other unobserved background covariates, which could have an effect on our results. In such cases, Rosenbaum (2010) has recommended to consider sensitivity analyses of how the Fisher-exact p-value would change, had the intervention assignment been plausibly different, see also Bind & Rubin (2020). Subject-matter knowledge on the probability of the binary exposure (i.e., smoking or air pollution) given the observed and unobserved background covariates should guide the plausible range of ``sensitivity'' p-values and the reason why they could deviate from the p-value calculated based on the assumed hypothetical intervention assignment. This idea provides material for an extension of the framework presented in this study.

2. The authors responded that the covariate adjustments method is unreliable, citing several references. However, the covariate adjustments method is another common approach used in causal inference (Pearl, 2000); the combination of this method with an inverse propensity scores weighting (known as the doubly robust estimator) is one of the most popular methods used in causal inference. Could the authors demonstrate its unreliability empirically? The estimates of the covariate adjustment method can also be given a causal interpretation under the same assumption of no unmeasured confounding effect. So, it is important to demonstrate matching, which discards a large portion of samples, is more reliable than the covariate adjustments method, which uses all samples.

The unreliability of regression adjustments in certain cases, as well as the appealing properties of using the Rubin Causal Model, have already been discussed extensively. Note that it is not our objective to convince the reader that one method is better than the other. We simply find the RCM compelling to examine the effects of environmental exposures on the gut microbiome. We tried to give more references to the standard regression modeling the observed data vs. the construction of hypothetical experiment literature using the potential outcome framework, and elaborate on our objective to rely on non-parametric methods as a first step to discoveries for this untapped research question in the Discussion p. 12:

> Second, the assumed assignment mechanism and underlying assumptions have to be clearly stated to obtain meaningful p-values. Standard approaches usually make strong assumptions (e.g., linearity), whose discussions are often neglected. Modeling the observed data and solely adjusting for confounders by including them in a regression, without a design stage, can be unreliable, especially when the pre-exposure covariates distributions of the control and intervention units are not similar. For instance, Cochran & Rubin (1973), Heckman et al. (1998), and Rubin (2001) have shown that regression models can estimate biased treatment effects

when the true relationship between the covariates and the outcome is not modeled accurately. Dehejia & Wahba (1999) have also shown that standard nonexperimental estimators such as regression are sensitive to the specification used in the regression. This is another reason why we opted for an inference method that does not rely on parametric assumptions.

3. Response to the authors' question about column titles of Table 1 (now Table 4):

In the Characteristics of Study Population section, a paragraph says that the number of matched pairs is 99 for the air pollution reduction experiment and 271 for the smoking prevention experiment. However, the sum of F and M is 271 in the left column (titled Air Pollution) and 99 in the right one (titled Smoking). I am not sure if only these numbers were switched or the column names were switched. I assumed the latter.

Thank you very much for carefully looking at the table. Indeed, the N (%) numbers in the Table were switched and have now been rectified.
